# TYK2 Promotes Immunosurveillance of Colorectal Cancer Liver Metastasis

**DOI:** 10.1158/0008-5472.CAN-24-4224

**Published:** 2026-01-02

**Authors:** Bernadette Mödl, Daniela Zwolanek, Katharina Schwertner, Dana Krauß, Stefan Moritsch, Irene Scharf, Anne-Sophie Ebner, Veronica Moreno-Viedma, Cristiano de Sa Fernandes, Philipp Novoszel, Martin Holcmann, Martina Hammer, Nunzia Matrone, Michaela Schlederer, Birgit Strobl, Emilio Casanova, Caroline Lassnig, Dietmar Herndler-Brandstetter, Lukas Kenner, Mathias Müller, Maria Sibilia, Robert Eferl

**Affiliations:** 1 Center for Cancer Research & Comprehensive Cancer Center (CCC), Medical University of Vienna, Vienna, Austria; 2 Department of Pathology, Medical University of Vienna, Vienna, Austria; 3 Institute for Animal Breeding and Genetics, University of Veterinary Medicine, Vienna, Austria; 4 Institute of Pharmacology, Center of Physiology and Pharmacology & Comprehensive Cancer Center (CCC), Medical University of Vienna, Vienna, Austria; 5 Ludwig Boltzmann Institute for Hematology and Oncology, Medical University of Vienna, Vienna, Austria

## Abstract

Colorectal cancer liver metastasis (CRLM) is a major clinical problem. The regulators of immunosurveillance of CRLM could hold potential for developing therapeutic strategies to prevent or treat metastasis. Here, using a murine colorectal cancer (CRC) organoid-based transplantation mode, we identified TYK2 as a key factor controlling CRLM. Evaluation of the effects of *Tyk2* deletion in different subsets of immune cells and in CRC cells demonstrated that TYK2 was not required in cancer cells, macrophages, NK cells, T cells, or Kupffer cells. Instead, TYK2 controlled CRLM via a dendritic cell-dependent mechanism that relied on MHC-I-mediated cross presentation of antigens to CD8^+^ T cells. Analysis of single-cell RNA sequencing data from primary CRC and CRLM revealed that TYK2 was predominantly expressed in a dendritic cell population destined to present antigens in tumor-draining lymph nodes. Treatment with the TYK2 inhibitor deucravacitinib, which is approved by the FDA for treating plaque psoriasis and is under clinical investigation for other autoimmune diseases, promoted CRLM. Together, these data demonstrate that TYK2 controls CRLM immunosurveillance, which should be carefully considered when treating patients with TYK2 inhibitors.

## Introduction

The Janus kinase-signal transducer and activator of transcription (JAK-STAT) pathway was originally described as a key mediator of antiviral defense (1,2). It was later linked to cancer development and is now considered a central cancer pathway as deregulation of JAK-STAT proteins has been observed in various cancer types (3,4). Activating mutations of TYK2 and formation of constitutively active fusion proteins are key cancer cell-intrinsic drivers of hematopoietic malignancies (5,6). In solid cancers, no activating TYK2 mutations have been detected, but increased expression has been observed in prostate, breast, cervical, ovarian and peripheral nerve sheath malignancies (7–11). Due to the lack of specific antibodies for TYK2 immunohistochemistry and the scarcity of *Tyk2* mRNA reads in single cell sequencing data, it remains to be demonstrated whether TYK2 expression is increased in cancer cells or cells of the tumor microenvironment. We have recently shown that TYK2 suppresses the expression of indolamine 2-3-deoxygenase 1 (Ido-1) in cancer cells, thereby promoting anti-cancer immunity of autochthonous colitis-associated CRC (12). Since autochthonous colorectal tumors of mice do not metastasize, it is currently unclear whether this tumor-suppressive function also applies to colorectal cancer metastasis.

In contrast to cancer cell-intrinsic functions, the role of TYK2 in the tumor immune microenvironment is much better defined. Several studies have demonstrated that TYK2 has essential functions in tumor immunosurveillance. TYK2 transduces signals from the anti-tumorigenic cytokines interferon α/β (IFN-α/β) and interleukin (IL)-12, resulting in production of IFN-γ and IL-15. The latter promote antigen presentation by dendritic cells (DCs) and increase the cytotoxicity of T and NK cells (13). Mouse models for hematopoietic malignancies and our data from the autochthonous CRC model suggest that TYK2-deficient immune cells exhibit impaired tumor immunosurveillance (12,14,15). Furthermore, the inherited *Tyk2* allele P1104A, which exhibits impaired kinase activity, was found in patients with solid tumors (lung, breast, stomach, colon, liver) and suggested to be a cancer-associated hypomorph. However, only 4 of 128 cancer samples carried this allele (16). A more recent GWAS study linked the same polymorphic *Tyk2* allele to lung cancer and non-Hodgkin lymphoma, but no significant association with CRC was found. It remains to be shown whether this *Tyk2* allele affects tumor immunosurveillance (17). A major problem for CRC patients is metastasis, which drastically shortens their life expectancy. The liver is the predominant metastatic site for CRC. This is favored by the immunotolerant hepatic state, which must prevent excessive immune responses to the antigens constantly delivered from the intestine (18). The hepatic events of CRLM consist of several dynamic phases. Disseminated cancer cells that invade the liver sinusoids via hemodynamics from the portal vein are retained by interactions with various cell adhesion molecules, such as E-selectin, on the liver sinusoidal endothelial cells. Here, cancer cells can be killed by hepatic immunity, which is primarily executed by Kupffer cells and natural killer cells. However, Kupffer cells also generate a pro-metastatic inflammatory state and produce pro-metastatic factors such as IL-6, HGF, VEGF, MMP-9 and MMP-14 (18). Surviving cancer cells invade the space of Disse, proliferate and form a metastatic vasculature. This phase is characterized by extensive remodeling of the extracellular matrix and orchestrated by various cell types, including stellate cells, Kupffer cells, neutrophils, hepatocytes and cancer-associated fibroblasts. Eventually, micrometastatic lesions may grow and form macrometastases with a stroma and typical immune infiltrates (18). The immunological events in immunosurveillance of CRLM, and in particular the role of TYK2 in this context, are largely unexplored.

JAK-STAT signaling plays a dual role in various pathologic conditions, including cancer and autoimmune diseases (19). The outcome depends on the cytokines involved, the corresponding activation of specific JAK-STAT combinations and the target cell type (19). For example, opposing roles of STAT1 and STAT3 have been described in several immune cell types such as macrophages, with IFN-γ-JAK1/JAK2-STAT1 promoting M1 polarization, while IL-10-JAK1/TYK2-STAT3 promotes M2 polarization (20). As a critical regulator of immune cell function, TYK2 became an interesting therapeutic target. TYK2 is activated by a narrower spectrum of cytokines than other JAKs such as JAK1, which could mitigate side effects of inhibitor treatment. Recently developed inhibitors bind to the regulatory pseudokinase domain and block TYK2 with high specificity, while leaving other JAKs unaffected (21,22). The first allosteric TYK2 inhibitor, deucravacitinib, has been approved by the FDA for the treatment of plaque psoriasis and is being used in clinical trials for other autoimmune diseases including inflammatory bowel disease (IBD).

Given the importance of TYK2 as therapeutic target in immune diseases, we investigated its functions in immunosurveillance of CRLM. Metastasis was induced with CRC organoids in different strains of mice with deletion of *Tyk2* in specific immune cell types. In addition, the impact of pharmacological TYK2 inhibition was assessed. Our data show that TYK2 plays a crucial role in immunosurveillance of CRLM and significantly prevents colonization of metastases in the liver. These results emphasize the importance of carefully considering the anti-metastatic functions of TYK2 when using TYK2 inhibitors in patients.

## Material and Methods

### Mice

TYK2^-/-^, TYK2^flox/flox^, TYK2^flox/flox^ Vav-Cre, TYK2^flox/flox^ CD4-Cre, TYK2^flox/flox^ CD11c-Cre, TYK2^flox/flox^ Ncr1-Cre and TYK2^flox/flox^ LysM-Cre mice have been described previously (12,23–25). TYK2^+/-^ mice were intercrossed to obtain TYK2^-/-^ and wild-type TYK2^+/+^ littermates. TYK2^flox/flox^ Vav-Cre, TYK2^flox/flox^ CD4-Cre, TYK2^flox/flox^ CD11c-Cre and TYK2^flox/flox^ Ncr1-Cre mice were crossed with TYK2^flox/flox^ mice to generate cre-negative TYK2^flox/flox^ control littermates and cre-positive TYK2^Δhem^, TYK2^ΔT^, TYK2^ΔDC^ and TYK2^ΔNK^ mice, respectively. TYK2^flox/+^ LysM-Cre mice were intercrossed to obtain TYK2^ΔM^ and TYK2^+/+^ LysM-Cre littermate controls. Mice were kept on a C57BL/6 background and housed at constant temperature, with 12 hours light/dark cycles under specific pathogen-free (SPF) conditions at the Core Facility Laboratory Animal Breeding and Husbandry (CFL) of the Medical University of Vienna and the Institute of Animal Breeding and Genetics of the University of Veterinary Medicine Vienna. Mouse experiments were performed with adult 8- to 12-week-old mice (weight between 20 and 30 grams). The allocation of experimental groups was based on the mouse genotypes without further bias. All experiments were approved by the Ethics Committee for Animal Experiments of the Medical University of Vienna. To select the optimal group size, a power analysis was conducted in collaboration with the statistical experts of the Ethics Committee. The experiments were conducted under an animal experiment license from the Austrian Federal Ministry of Science and Research in accordance with Austrian and European laws and with the general regulation specified by the Good Scientific Practices guidelines of the Medical University of Vienna.

### Mouse genotyping by polymerase chain reaction (PCR)

PCR on isolated genomic DNA was performed using the DreamTaq Green DNA Polymerase Kit (EP0714, Thermo Scientific™) with the following primers (5’>3’): TYK2: gcaagcctgggttacatgag, tggactggaacttgtgagga. Vav-Cre: tcagagtgaaggacatctcccgcacc, gtggcagaaggggcagccacaccatt. CD4-Cre: cggtcgatgcaacgagtgatgagg, ccagagacggaaatccatcgctcg. CD11c-Cre: acttggcagctgtctccaag, gcgaacatcttcaggttctg. Ncr1-Cre: tgatgctgggtttggcccagatg, atgcggtgggctctatggcttctg. LysM-Cre: gcattgcagactagctaaaggcag, gtcggccaggctgactccatag, cccagaaatgccagattacg.

### Isolation of AKP organoids

AKP organoids were isolated from spontaneously developing polyps of male Villin-Flp, Apc^Min/+^ (A), p53^FRT^ (P), FSF-Kras^G12D/+^ (K) mice with an additional reporter gene, in which firefly luciferase and eGFP are activated upon Flp recombination (R26Dual) (26). The expression of firefly luciferase and eGFP allowed the monitoring of the tumor development by bioluminescence *in vivo* imaging and in histological samples. In detail, polyps were collected from excised and PBS-flushed colons and minced into smaller pieces. This material was enzymatically digested with 2 mg/mL collagenase I (07902, Stemcell Technologies), 50 μg/ml gentamicine (G1264, Sigma-Aldrich), 1 mg/ml DNase (DN25, Sigma-Aldrich) in advanced DMEM/F12 (12634028, Thermo Scientific) for 40 min at 37°C with vigorous pipetting every 5 to 10 min. Digested polyp fragments were washed with advanced DMEM/F12 with 20% FBS (F9665, Sigma-Aldrich) and strained (40 μm). Small tissue fragments were collected by a low centrifugation force and decreased deceleration (100×g, 10 min, acceleration 9, deceleration 6), keeping single cells in suspension and crypts in the pellet. Isolated tissue fragments were cultivated in Geltrex™ (12053569, Thermo Fisher Scientific) as described below. 10 μM Y27632 (SCM075, Merck) were added to the standard organoid medium used for cultivation for the first 5 days. Single cell clones were generated, genotyped and tested for their metastatic capacity *in vivo* using splenic injections.

### Generation of AKPT organoids

*Tyk2* (T) was deleted from AKP CRC organoids by CRISPR/Cas9 to generate AKPT organoids as previously described (27). In brief, four *Tyk2*-specific sgRNAs were cloned into the 4-gRNA-concatemer plasmid (84881, Addgene, RRID:Addgene_84881). Two different approaches were used targeting either exon 1, 5, 14 and 19 or 3, 6, 19 and 21 of *Tyk2*. sgRNAs used are listed in the table below. Sanger sequencing was performed to ensure insertion of all four sgRNAs in isolated plasmids. The following sequencing primers were used:

mTYK2_ex1_cas1_F: caccggGGCAAGTGGCGCGTAGGCGGgt,

mTYK2_ex1_cas1_R: taaaacCCGCCTACGCGCCACTTGCCcc,

mTYK2_ex6_cas1_F: caccggGGCCAGGCCGGAAGGCCCGCgt,

mTYK2_ex6_cas1_R: taaaacGCGGGCCTTCCGGCCTGGCCcc,

mTYK2_ex3_cas2_F: accggATCCACATCGCACACAAAGTg,

mTYK2_ex3_cas2_R: aaaacACTTTGTGTGCGATGTGGATc,

mTYK2_ex5_cas2_F: accggGCGGGACCTGTCTAGCGAGGg,

mTYK2_ex5_cas2_R: aaaacCCTCGCTAGACAGGTCCCGCc,

mTYK2_ex19_cas3_F: ccggGTTCGTGGTACAGCGTCCGC,

mTYK2_ex19_cas3_R: aaacGCGGACGCTGTACCACGAAC,

mTYK2_ex14_cas4_F: acaccggGTGTGGTTACGGCGACAGAGgtt,

mTYK2_ex14_cas4_R: ctaaaacCTCTGTCGCCGTAACCACACccg,

mTYK2_ex21_cas4_F: acaccggGTCCAGCAGCACGTTGCGCGgtt,

mTYK2_ex21_cas4_R: ctaaaacCGCGCAACGTGCTGCTGGACccg,

Enzymatically digested small AKP organoid fragments were co-transfected with the newly generated *Tyk2*-specific sgRNA plasmid and the lentiCas9-EGFP plasmid (63592, Addgene, RRID:Addgene_63592) using the NEPA21 electroporator (Nepa Gene). Successful transfection was confirmed by fluorescence microscopy and puromycin resistance cassette allowed for antibiotic selection of plasmid containing cells. Single cell clones were picked and *Tyk2* deletion was confirmed by qPCR and Western blot analysis, described below.

### Cultivation of organoids and processing for injection

AKP and AKPT organoids were cultured three-dimensionally in droplets of basement membrane matrix (Geltrex™, 12053569, Thermo Fisher Scientific) diluted with advanced DMEM/F-12 (11550446, Thermo Fisher Scientific). The droplets were incubated at 37 °C for 20 minutes and then overlaid with DMEM/F12 supplemented with 10% FBS (F9665, Sigma-Aldrich), 1 × GlutaMAX (2 mM L-alanyl-L-glutamine dipeptide, 11574466, Thermo Fisher Scientific), 10 mM HEPES (15630080, Thermo Fisher Scientific), 1 × B 27 (11530536, Thermo Fisher Scientific), 1 × N 2 (11520536, Thermo Fisher Scientific), 50 ng/ml mEGF (10564614, Thermo Fisher Scientific), 2 μM galunisertib (TGFβRI inhibitor, 6956/5, R&D Systems), 1 mM N-acetyl-L-cysteine (A9165-5G, Sigma) and mixed 1:1 with a supernatant form L-WRN cells containing Wnt3a, R-spondin-3 and noggin (28,29). For surgery, organoids were mechanically and enzymatically dissociated into single cells by vigorous pipetting in trypsin (T4049, Sigma-Aldrich), followed by straining the cells through a 40 μM mesh. Single organoid cells were re-suspended in HBSS/ 0.25% BSA in a concentration of 500,000 cells in 10 μl.

### Intrasplenic/intraportal injection of organoids and deucravacitinib treatment

Since organoids were obtained from male mice, all metastasis experiments with organoids were performed with male hosts to avoid sex-based rejection. According to Austrian law mice were anesthetized using ketamine and xylazine. After shaving and disinfecting the left side of the abdomen with povidone-iodine, a small incision was made into the peritoneum and the spleen was exposed. 500,000 organoid single cells in 10 μl HBSS/0.25% BSA were injected into the spleen using a Hamilton syringe. When bleeding stopped, the spleen was pushed back into the abdominal cavity, the incision was cleaned with sterile PBS and properly closed with stitches and metal staples. For portal vein injections, a 3-4 cm long incision was made at the mid of the abdomen. Intestines were pushed to the right side and covered by PBS-soaked sterile tissues to expose the portal vein. Using a Hamilton syringe 250,000 organoid single cells in 10 μl HBSS/0.25% BSA were injected and bleeding was stopped by using alginate coated cotton pads. Subsequently, the incision was properly cleaned and closed. To inhibit TYK2 systemically, mice were orally gavaged with deucravacitinib (HY-117287, MCE) at a dose of 30 mg/kg daily. Deucravacitinib was prepared in a suspension of 5% DMSO (A36720, Applichem), 5% Tween80 (HY-Y1891, MCE), 50% NaCl and 40% PEG300 (HY-Y0873, MCE) and 150 μl were administered to each mouse. Treatment of mice started one day before organoid injection to ensure stable TYK2 inhibition at the time of injection (22).

### MC38 cell culture and intraperitoneal injection

The female murine adenocarcinoma cell line MC38 was obtained from ATCC (RRID:CVCL_B288). Cell authentication was performed using STR profiling. Cells were grown in DMEM (41965039, Thermo Fisher Scientific), supplemented with 10% FBS (F9665, Sigma-Aldrich), 1% non-essential amino acids (11140035, Gibco), 1 mM Sodium Pyruvate (P2256, Sigma) and 2 mM Glutamine (A2916801, Thermo Fisher Scientific) and transfected with a GFP expression vector as described previously (30). The cells were regularly tested for mycoplasma contamination upon freezing of stocks using nested PCR of heat-inactivated cell culture supernatants. No mycoplasma testing or cell authentication was performed immediately before intraperitoneal injection. For intraperitoneal injections, MC38 cells (passage 2 post-thawing) were dissociated with Trypsin-EDTA (25200056, Thermo Fisher Scientific), washed and resuspended in HBSS/0.25% BSA (A9418, Sigma-Aldrich). 10^6^ cells in 100 μl HBSS/0.25% BSA were injected into the peritoneum of each mouse. Mice were sacrificed after 3 weeks, and peritoneal tumors were quantified.

### *In vivo* bioluminescence imaging (BLI)

*In vivo* BLI was performed using an IVIS Spectrum system (PerkinElmer, Santa Clara, CA). Mice received an intraperitoneal injection of D-luciferin (150mg/kg, Biovision, StayBrite™ D-Luciferin, sodium salt) 10 min prior to imaging. During imaging, animals were anesthetized with 2% isoflurane (50019100, Zoetis) delivered via nose cone and maintained at a constant temperature on the IVIS warming stage. Bioluminescent signals were captured at a 1.5 cm height with medium binning and exposure times ranging from 2 to 5 min, optimized for each mouse model. Imaging was conducted three weeks post organoid injection.

### Histology and immunohistochemistry

2 h before necropsy 0.25 μg BrdU (5-bromo-2’-deoxyuridine, P5002, Sigma-Aldrich) per g body weight was injected i.p. for sufficient BrdU incorporation. Livers were harvested 3-7 weeks after splenic organoid injection and fixed in 4% formaldehyde (P087, Carl Roth) and embedded in paraffin. 2.5 μm thick sections were deparaffinized in xylene and rehydrated in decreasing ethanol series. Immunohistochemistry (IHC) and hematoxylin & eosin (H&E) staining were performed using standard procedures (31). In brief, for IHC, antigen retrieval was performed for one hour in a steamer either using a pH6.0 citrate buffer or pH9.0 Tris-EDTA buffer. Subsequent blocking was performed by using hydrogen peroxide, avidin, biotin and the Mouse to Mouse HRP Staining System (MTM003-IFU, ScyTec). Slides were stained using antibodies for GFP (D5.1, 2956, Cell Signaling Technology, RRID:AB_1196615), PDGFR (28E1, 3169, Cell Signaling, RRID:AB_2162497), BrdU (BU1/75 (ICR1), ab6326, Abcam, RRID:AB_305426), Ki67 (D3B5, 12202, Cell Signaling Technology, RRID:AB_2620142), CD8a (D4W2Z, 98941, Cell Signaling Technology, RRID:AB_2756376), CD4 (D7D2Z, 25229, Cell Signaling Technology, RRID:AB_2798898), F4/80 (D2S9R, 70076, Cell Signaling Technology, RRID:AB_2799771), CD11c (D1V9Y, 97585, Cell Signaling Technology, RRID:AB_2800282), NK1.1/CD161 (E6Y9G, 39197, Cell Signaling Technology, RRID:AB_2892989) and Endomucin (V.7C7, 14-5851-82, eBioscience, RRID:AB_891527) in 1% BSA. Stainings were developed with AEC substrate chromogen (K346, Dako) and counterstained with hematoxylin. For H&E staining, sections were incubated in hematoxylin for 10 min, dehydrated in an increasing ethanol series, and stained with eosin for 2 min. Histological analysis was performed by employing HALO® (Indica labs), Definiens Tissue Studio®, ImageJ and CaseViewer (3DHISTECH Ltd). Tumor load was calculated as the ratio between tumor area and total tissue area. The number of tumor lesions per area is referred to as tumor multiplicity.

### Immunofluorescence staining

For immunofluorescence staining, antigen retrieval was performed at pH9.0. Simultanous blocking and permeabilization was carried out with 0.5% Saponin (47036, Sigma-Aldrich), 2% goat serum (S-1000-20, Vector Laboratories), 2.5% methanol in PBS. Sections were incubated with an antibody for CD8a (D4W2Z, 98941, Cell Signaling Technology, RRID:AB_2756376) followed by staining with anti-rabbit-Alexa Fluor 488 (A11034, Thermo Fisher Scientific, RRID:AB_2576217). Subsequent staining for PD-1 was performed (EPR20665, ab214421, Abcam, RRID:AB_2941806), and nuclei were stained with DAPI (10236276001, Roche). Slides were scanned using an VS200 Slidescanner (Evident).

### Quantitative PCR

The TRIzol Reagent (12034977, Thermo Fisher Scientific) was used to isolate RNA from tissues and cells. RNA was reverse transcribed using the QuantiTect Reverse Transcription Kit (205314, Qiagen). For low cell numbers, RNA was isolated using RNeasy Protect Mini Kit (74124, Qiagen) and reverse transcribed with SuperScript™ IV Reverse Transcriptase (15307696, Thermo Fisher Scientific). qPCR was performed using the GoTaq qPCR Master Mix (Promega) and CFX96 Real-Time System (Biorad) with the following primers (5’>3’): *Tyk2*: (1) cccacaggatgcttgatggt, cgactttgtgtgcgatgtgg, (2) tgagcagggatggtatgttgg, ggtggatctcctcctcgcta, (3) aaagtcggcatcactccacc, caccggtatacagctggctc. *IFN-α*: ctactggccaacctgctctc, ctgctgggcatccaccttc. *IFN-γ*: cggcacagtcattgaaagcc, caagacttcaaagagtctgagg. *IL-15*: gcaatgaactgctttctcctgg, cctccagctcctcacattcc. *CD86*: gatgcaccatgggcttggc, ctgtgcccaaatagtgctcgtac. *Gapdh*: tgtttgtgatgggtgtg, tacttggcaggtttctc. Tbp: ggggagctgtgatgtgaagt, ccaggaaataattctggctcat. Results were calculated using the delta Ct method. Relative quantification was achieved by normalizing to the expression values of the *Gapdh* or *Tbp* housekeeping gene.

### Western blot analyis

Western blot analysis was performed as previously described (32). Antibodies for TYK2 (33), pSTAT1 (9167, Cell Signaling Technology, RRID:AB_561284), β-actin (3700, Cell Signaling Technology, RRID:AB_2242334) and Vinculin (4650, Cell Signaling Technology, RRID:AB_10559207) were used.

### *In vitro* differentiation of macrophages from bone marrow

Mice were sacrificed and bone marrow was isolated from the femur and tibia by flushing the bones with DMEM. Erythrocytes were lysed prior to plating of bone marrow cells. For bone marrow-derived macrophages (BMDMs), stem cells were differentiated into macrophages by cultivation in conditioned medium of L929 cells (34), diluted 1:5 with DMEM. Medium was changed every second day and differentiation was confirmed after one week by flow cytometry using antibodies for CD11b (101259, BioLegend, RRID:AB_2566568) and F4/80 (157303, BioLegend, RRID:AB_2832546).

### Flow cytometry of immune infiltrates in liver metastases

Livers were harvested and metastatic nodules were excised, minced, and digested at 37°C for 45 minutes with collagenase IV (10780004, Sigma-Aldrich). Digested tissue was strained through a 70 μm mesh to yield a single cell suspension. Erythrocytes were lysed using ACK lysis buffer and fluorochrome-conjugated antibodies were used to detect mouse surface proteins. CD45 was used as a marker for leukocytes (103153, BioLegend, RRID:AB_2572115). Lymphocyte subsets were characterized using antibodies for CD3 (100306, BioLegend, RRID:AB_312671), CD4 (25-0042-82, eBioscience, RRID:AB_469578), CD8 (100737, BioLegend, RRID:AB_10897101) and NK1.1 (108739, BioLegend, RRID:AB_2562273). Myeloid subsets were identified using antibodies for CD103 (121420, BioLegend, RRID:AB_10714791), CD11b (101259, BioLegend, RRID:AB_2566568), CD11c (117316, BioLegend, RRID:AB_493566), CD64 (139304, BioLegend, RRID:AB_10612740), F4/80 (123133, BioLegend, RRID:AB_2562305), MHC-II (107614, BioLegend, RRID:AB_313329), Ly6C (128017, BioLegend, RRID:AB_1732093) and Ly6G (127627, BioLegend, RRID:AB_10897944). Zombie Aqua (423103, BioLegend) and 7-AAD (420404, BioLegend) were used to exclude dead cells. Data acquisition was performed using and LSRFortessa X-20 flow cytometer and FACSDiva software (BD Biosciences). Flow cytometry data were analyzed using Flowjo software (BD Biosciences).

### Isolation of Kupffer cells

Murine Kupffer cells (KC) were enriched following liver perfusion (LP) as previously reported (35). The perfusion system was designed as a closed circuit with a roller pump to provide a constant flow rate. Livers were perfused at 7 ml/min via the portal vein using a liver perfusion buffer (17701038, Thermo Fisher Scientific) to flush out blood, followed by further liver digestion medium (17703034, Thermo Fisher Scientific). The digested liver tissues were ground and passed into a 50 ml tube through a 70 μm cell strainer. The mixture was centrifuged at 50 g for 4 min at 4°C to remove hepatocytes, the supernatant was collected and washed three times in 0,1% BSA-PBS (B-PBS). Non-parenchymal cells (NPCs) in the supernatant were pelleted by high-speed centrifugation, resuspended in 10 ml density gradients solution (17,6% OptiPrep D1556, Sigma-Aldrich), followed by sequential layering of 10 ml 8,2% Optiprep solution and 4 ml B-PBS. KC were located in the interface between the 8,2% and 17,6% OptiPrep layers and plated onto uncoated cell culture dishes in RPMI-1640 (22400-089, Gibco), supplemented with 10% FCS (F9665, Sigma-Aldrich), 2 mM glutamine (G7513, Sigma-Aldrich) and 1% penicillin–streptomycin (P4333, Sigma-Aldrich) for 24 hours.

### Cell sorting

TYK2 deletion in different Cre mouse lines was confirmed by isolating cells from spleens and cell sorting of desired populations. In brief, mice were sacrificed, spleens were harvested and strained (70 μm pore size) to obtain a single cell suspension. Erythrocytes were lysed and immune cells were stained using fluorochrome-conjugated antibodies for CD3 (100306, BioLegend, RRID:AB_312671), CD8 (100737, BioLegend, RRID:AB_10897101) and CD4 (25-0042-82, eBioscience, RRID:AB_469578) for T cell sorting. To obtain pure NK cells, exclusion stainings for CD11b (101256, BioLegend, RRID:AB_2563648), MHC-II (107608, BioLegend, RRID:AB_313323), B220 (103208, BioLegend, RRID:AB_312993), CD3 (100306, BioLegend, RRID:AB_312671) and Ly6G (127612, BioLegend, RRID:AB_2251161) were performed, whereas NK1.1 (108715, BioLegend, RRID:AB_493591) was used as a positive marker for NK cells. To isolate DCs, NK1.1 (108706, BioLegend, RRID:AB_313393), CD19 (115505, BioLegend, RRID:AB_313640), CD3 (100306, BioLegend, RRID:AB_312671) and Gr-1 (108406, BioLegend, RRID:AB_313371) antibodies were used as negative markers. Antibodies for CD11c (117353, BioLegend, RRID:AB_2686978), BST2 (127104, BioLegend, RRID:AB_1953283) and MHC-II (107620, BioLegend, RRID:AB_493527) were used to sort DCs. Cell sorting was performed using a FACSMelody Cell Sorter (BD Biosciences).

### Adoptive T cell transfer

Spleens and skin lymph nodes of OT-I and OT-II mice (CD45.1) were harvested, cells were strained (70 μm pore size) and erythrocytes were lysed. CD4^+^ and CD8^+^ T cells were isolated using magnetic-activated cell sorting. Biotinylated antibodies for B220 (553086, BD Biosciences, RRID:AB_394616), CD11b (101204, BioLegend, RRID:AB_312787), CD11c (117304, BioLegend, RRID:AB_313773), NK1.1 (108704, BioLegend, RRID:AB_313391), TER119 (116204, BioLegend, RRID:AB_313705), Gr-1 (108404, BioLegend, RRID:AB_313369), CD19 (553784, BD Biosciences, RRID:AB_395048) and CD4 (100404, BioLegend, RRID:AB_312689) or CD8 (100704, BioLegend, RRID:AB_312743) were used to negatively select T cells. Isolated T cells were labelled with CellTraceTM Violet (CTV, C34571, Thermo Fisher Scientific) to monitor proliferation. On day 1, one million cells were injected into the tail vein of TYK2 KO mice (CD45.2). 1 mg of ovalbumin was intraperitoneally injected on day 2. On day 5, spleens were harvested and analyzed by flow cytometry using antibodies for CD45.1 (110705, BioLegend, RRID:AB_313494), CD45.2 (109851, BioLegend, RRID:AB_2629722), CD8 (100759, BioLegend, RRID:AB_2563510) and CD4 (100469, BioLegend, RRID:AB_2783035).

### *Ex vivo* DC stimulation

Spleens were minced and digested in PBS with Ca^2+^ and Mg^2+^ supplemented with 100 μg/ml Liberase (5401127001, Sigma-Aldrich) and 100 μg/ml DNase I (DN25, Sigma-Aldrich) at 37 °C for 30 min. Erythrocytes were lysed and cells were filtered through a 70-μm cell strainer. Splenic DCs were pre-enriched (> 50%) using the EasySep™ Mouse Pan-DC Enrichment Kit II (19863, Stemcell Technologies) according to manufacturer's instructions. The isolated cell fraction was then sorted on a BD FACSMelody™ Cell sorter for B220^-^CD19^-^CD11c^+^MHC-II^+^ cells (> 90%). Sorted DCs were re-plated in a cell culture medium containing RPMI-1640 (22400-089, Gibco) supplemented with 10% FCS (F9665, Sigma-Aldrich), 1% Penicillin– Streptomycin (P4333, Sigma-Aldrich), 1% glutamine (G7513, Sigma-Aldrich), 1% non-essential amino acids (11140035, Thermo Fisher Scientific), 0.1% β-mercaptoethanol (31350-010, Gibco), and 80 ng/ml murine FLT3L (250-31L, Prepotec). DCs were overnight stimulated with 5 μg/ml poly I:C (HMW, tlrl-pic, InvivoGen). Subsequently, cells were stained with the following fluorescently labelled antibodies: CD11c (117308, BioLegend, RRID:AB_313777), CD40 (124614, BioLegend, RRID:AB_1134069), CD80 (104706, BioLegend, RRID:AB_313127), CD86 (105014, BioLegend, RRID:AB_439783), CD274 (571067, BD Biosciences, RRID:AB_3686206), and MHC-II (107641, BioLegend, RRID:AB_2565975). Labelled cells were recorded on a LSR Fortessa flow cytometer and evaluated with the FACSDiva software (BD Biosciences).

### Kaplan-meier plotter and human single cell RNA expression data analysis

The Kaplan-meier plotter (https://kmplot.com/analysis/) was used to evaluate the impact of TYK2 expression on overall survival of colon cancer patients. The patients were split into low and high TYK2 expressers using the best cutoff threshold. Publicly available scRNA sequencing data were accessed from the Gene Expression Omnibus (GEO) database under the accession number GSE225857 (36). Provided raw count matrix was filtered and normalized according to authors previous analysis description, using the most recent version of *Seurat* (version 5.1.0) (37). Provided cell and sample annotations were utilized to analyze subsets of different immune cells. All processing was performed in R version 4.4.3.

### Bulk RNA Sequencing

RNA was isolated from liver tissue, metastases and liver tissue adjacent to metastases using the TRIzol Reagent (12034977, Thermo Fisher Scientific). RNA quality and integrity was assessed using the Agient2100, LabChip GX (Perkin Elmer). VAHTS Universal V8 RNA-seq Library Prep Kit for Illumina NR605 was used to construct the mRNA libraries according to the protocol provided by Vazyme. Sequencing was performed on the Novaseq X platform (Illumina). RNA quality assessment, library preparation and sequencing were performed by Biomarker Technologies. Data preprocessing and alignment to the mouse genome GRCm38/mm10 of fastq files were performed using the kallisto pipeline (38). Gene-level counts for each tissue were normalized by library size using DESeq2’s median-of-ratios method (39). Genes with low counts were filtered prior to normalization. Data processing was performed with RStudio version 4.3.1.

### Statistics

The normality of the data distribution was tested by Kolmogorov-Smirnov or D'Agostino-Pearson and statistical tests were performed accordingly. Comparisons of two groups were calculated with unpaired Student’s t-test or Mann-Whitney U test. For more than two groups one-way Analysis of Variance (ANOVA) and Tukey’s multiple comparison test, Bonferroni’s post-hoc test or Kruskal-Wallis test and Dunn’s post-hoc test were used. For the negative correlation analyses between immune infiltration and metastatic burden, the immune infiltration data sets showed non-normal distributions and the Spearman correlation coefficient was calculated. All analyses were performed using GraphPad Prism 8 software. A p value of < 0.05 was considered significant.

## Results

### TYK2 is essential for immunosurveillance of CRLM

The intrasplenic injection model of liver metastasis and CRC organoids, isolated from genetically-induced colorectal polyps of mice with mutations in Apc, Kras and p53 (AKP), were used to investigate functions of TYK2 in CRLM (26). The AKP organoids expressed green fluorescent protein (GFP) and luciferase (40) to enable non-invasive tracking of metastases *in vivo*. The organoids were additionally manipulated with CRISPR/Cas9 to generate AKPT organoids with deletion of *Tyk2* (Supplementary Figure 1a, b). To investigate cancer cell-intrinsic functions of TYK2 in CRLM, three independent AKP and AKPT organoid clones were injected into the spleen of C57BL/6 host mice. Corresponding in vivo imaging systems (IVIS) data showed a similar tumor burden of host mice with AKP and AKPT organoids (Supplementary Figure 1c), which was confirmed by macroscopic inspection of livers four weeks post injection (Supplementary Figure 1d). For microscopic analysis, liver sections were stained for GFP and with H&E (Supplementary Figure 1e). The metastatic burden was quantified by calculating the liver-to-body-weight ratio and histomorphometry of metastases on microscopic images (Supplementary Figure 1f, g). These analyses confirmed similar metastatic burden of host mice with AKP and AKPT organoids, suggesting that TYK2 has no cancer-intrinsic function in this CRLM model.

**Figure 1:**
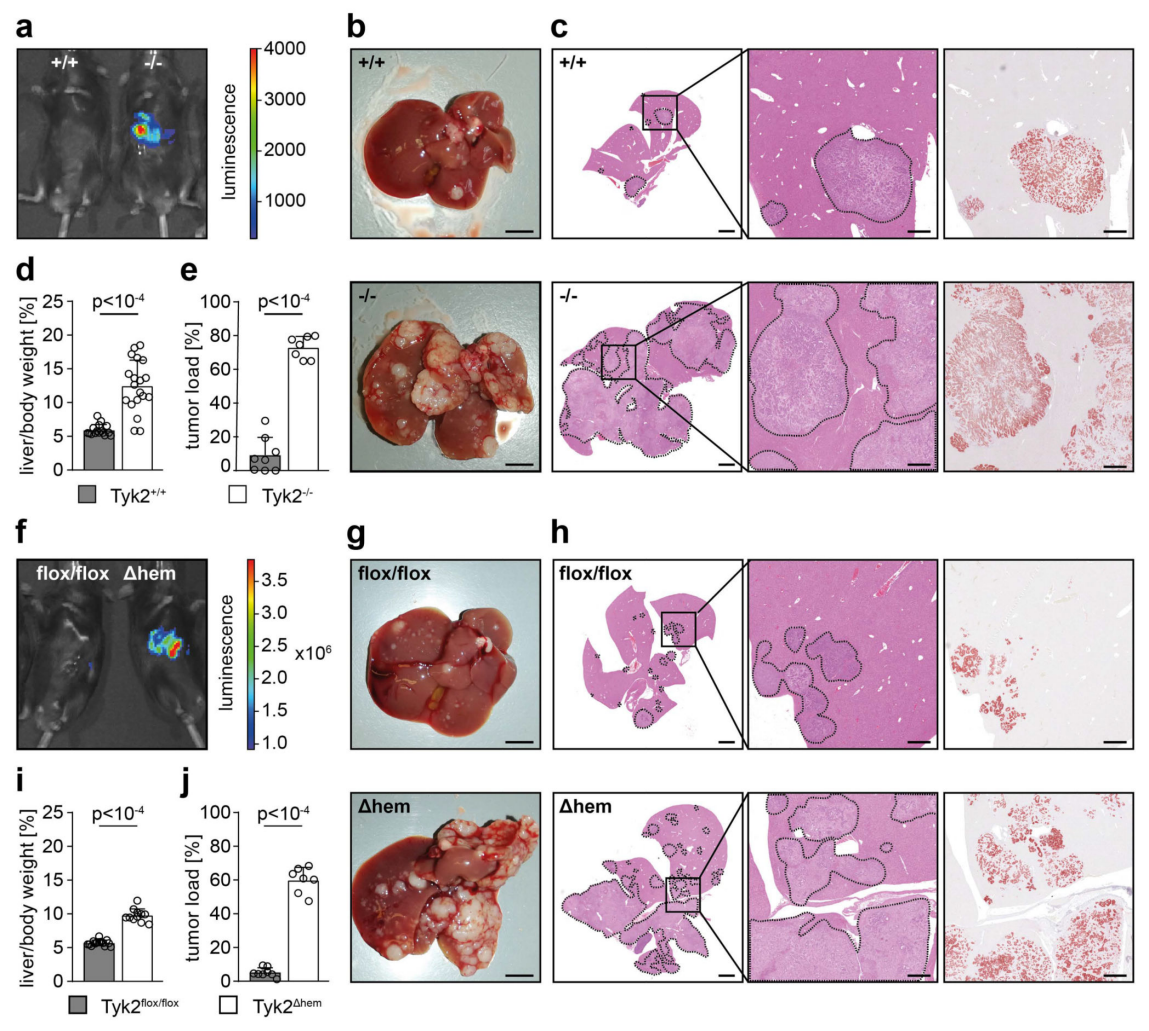
TYK2 deficiency in host mice promotes liver metastasis of AKP organoids. (**a**) Representative IVIS images of TYK2^+/+^ (+/+) and TYK2^-/-^ (-/-) host mice, 3 weeks after intrasplenic injection of AKP organoids. (**b**) Macroscopic images of livers of TYK2^+/+^ (+/+) and TYK2^-/-^ (-/-) host mice, 4 weeks after intrasplenic injection of AKP organoids. Scale bar = 5 mm. (**c**) H&E and GFP staining of liver sections of TYK2^+/+^ (+/+, upper images) and TYK2^-/-^ (-/-, bottom images) host mice, 4 weeks after intrasplenic injection of AKP organoids. The images in the center represent higher magnifications of the images on the left, with the square indicating the magnified region. Dashed lines mark metastatic lesions used to quantify the tumor load shown in (e). The images on the right show immunohistochemical GFP staining of consecutive sections. Tumor cells are red. Scale bar = 2 mm for the images on the left and 500 μm for the images in the center and on the right. (**d**) Liver-to-body weight ratio of TYK2^+/+^ and TYK2^-/-^ host mice, 4 weeks after intrasplenic injection of AKP organoids. (**e**) Histomorphometric quantification of the tumor load (% of tumor area to total tissue area) of TYK2^+/+^ and TYK2^-/-^ host mice, 4 weeks after intrasplenic injection of AKP organoids. (**f**) Representative IVIS images of TYK2^flox/flox^ (flox/flox) and TYK2^Δhem^ (Δhem) host mice, 3 weeks after intrasplenic injection of AKP organoids. (**g**) Macroscopic images of livers of TYK2^flox/flox^ (flox/flox) and TYK2^Δhem^ (Δhem) host mice, 4 weeks after intrasplenic injection of AKP organoids. Scale bar = 5 mm. (**h**) H&E and GFP staining of liver sections of TYK2^flox/flox^ (flox/flox, upper images) and TYK2^Δhem^ (Δhem, bottom images) host mice, 4 weeks after intrasplenic injection of AKP organoids. The images in the center represent higher magnifications of the images on the left, with the square indicating the magnified region. Dashed lines mark metastatic lesions used to quantify the tumor load shown in (j). The images on the right show immunohistochemical GFP staining of consecutive sections. Tumor cells are red. Scale bar = 2 mm for the images on the left and 500 μm for the images in the center and on the right. (**i**) Liver-to-body weight ratio of TYK2^flox/flox^ and TYK2^Δhem^ host mice, 4 weeks after intrasplenic injection of AKP organoids. (**j**) Histomorphometric quantification of the tumor load (% of tumor area to total tissue area) of TYK2^flox/flox^ and TYK2^Δhem^ host mice, 4 weeks after intrasplenic injection of AKP organoids. Bar diagrams represent mean values +/- SEM with each data point representing a mouse. Case Viewer, QuPath and Halo software were used for histomorphometry. Statistical analysis was performed using unpaired Student’s t-test. p values are indicated.

Intrasplenic injection of AKP organoids into TYK2-deficient (TYK2^-/-^) host mice was used to investigate cancer cell-extrinsic functions of TYK2. IVIS data showed a strongly increased tumor burden in TYK2^-/-^ host mice compared to littermate control host mice (TYK2^+/+^) three weeks post injection (Figure 1a), which was confirmed by macroscopic inspection of livers four weeks post injection (Figure 1b). The increased metastatic burden was also confirmed by microscopic inspection of liver sections, stained with H&E and for GFP (Figure 1c), as well as calculation of the liver-to-body-weight ratio and histomorphometry of metastases on microscopic images (Figure 1d, e). We hypothesized that the increased metastatic burden in TYK2^-/-^ host mice is due to a defect in immune functions leading to impaired tumor immunosurveillance. Therefore, the AKP organoids were injected into the spleen of TYK2^Δhem^ (TYK2^flox/flox^ Vav-Cre) host mice with specific deletion of *Tyk2* in hematopoietic cells. The greatly increased metastatic burden in TYK2^-/-^ host mice was phenocopied in TYK2^Δhem^ host mice (Figure 1f-j), suggesting that TYK2 signaling in immune cells limits metastatic growth. Furthermore, AKP organoids were injected into the portal vein of mice to exclude an influence of the intrasplenic immune environment on CRLM. The alternative injection route resulted in a similarly increased metastatic burden as the intrasplenic route in TYK2^-/-^ (Supplementary Figure 2a-e) and TYK2^Δhem^ host mice (Supplementary Figure 2f-j).

**Figure 2:**
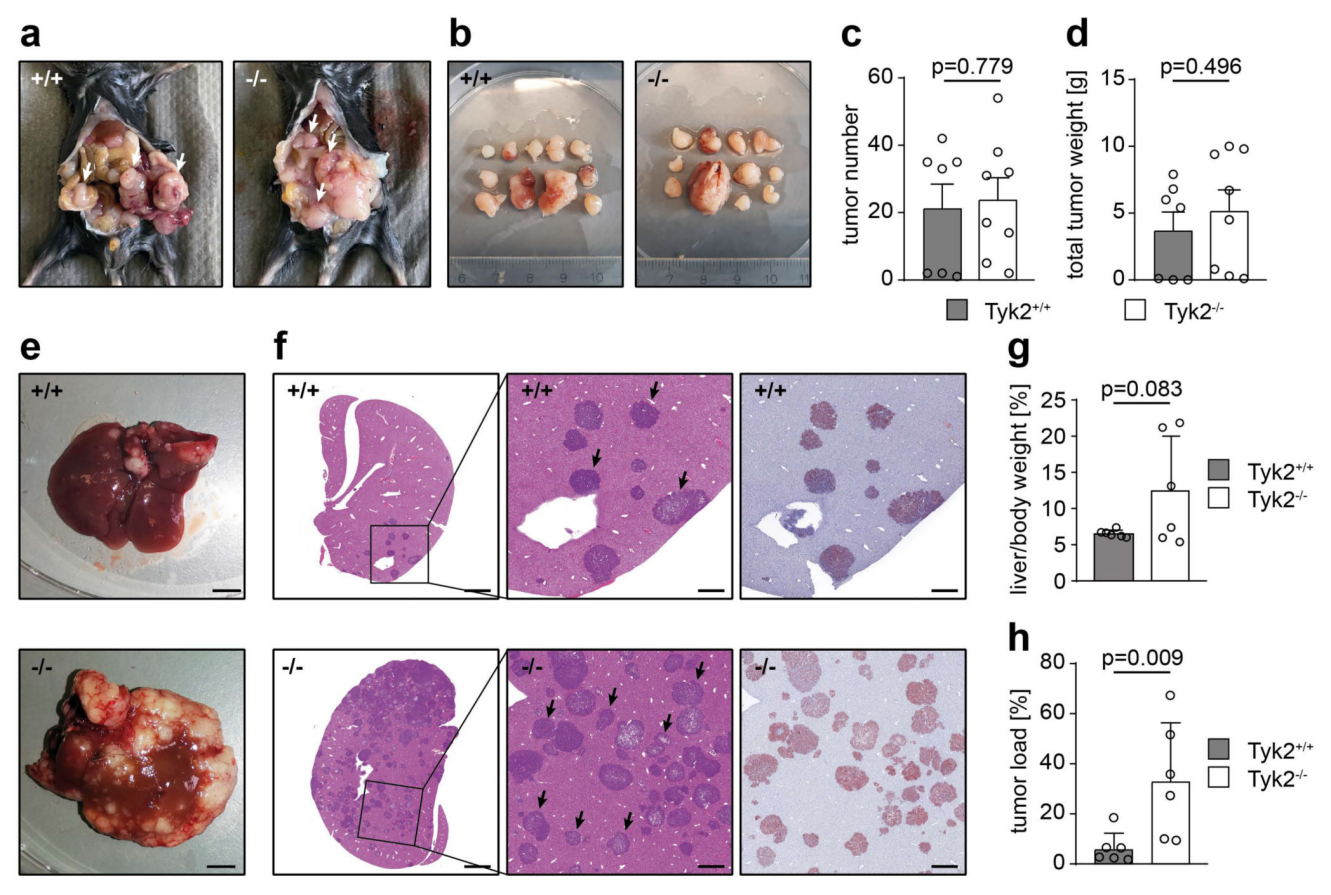
TYK2 deficiency in host mice promotes liver metastasis of MC38 cells but not peritoneal metastasis. (**a**) Macroscopic images of TYK2^+/+^ (+/+) and TYK2^-/-^ (-/-) host mice with peritoneal metastases, 3 weeks after intraperitoneal injection of syngeneic MC38 CRC cells. Tumors are indicated by arrows. (**b**) Isolated peritoneal metastases from TYK2^+/+^ (+/+) and TYK2^-/-^ (-/-) host mice, 3 weeks after intraperitoneal injection of syngeneic MC38 CRC cells. The scale is indicated by the ruler in cm. (**c**) Tumor numbers per mouse in TYK2^+/+^ (+/+) and TYK2^-/-^ (-/-) host mice, 3 weeks after intraperitoneal injection of syngeneic MC38 CRC cells. (**d**) Total tumor weight of metastases in TYK2^+/+^ (+/+) and TYK2^-/-^ (-/-) host mice, 3 weeks after intraperitoneal injection of syngeneic MC38 CRC cells. (**e**) Macroscopic images of livers of TYK2^+/+^ (+/+) and TYK2^-/-^ (-/-) host mice, 4 weeks after intrasplenic injection of syngeneic MC38 CRC cells. Scale bar = 5 mm. (**f**) H&E and GFP staining of liver sections of TYK2^+/+^ (+/+, upper images) and TYK2^-/-^ (-/-, bottom images) host mice, 4 weeks after intrasplenic injection of syngeneic MC38 CRC cells. The images in the center represent higher magnifications of the images on the left, with the square indicating the magnified region. Arrows mark metastatic lesions used to quantify the tumor load shown in (h). The images on the right show immunohistochemical GFP staining of consecutive sections. Tumor cells are red. Scale bar = 2 mm for the images on the left and 500 μm for the images in the center and on the right. (**g**) Liver-to-body weight ratio of TYK2^+/+^ and TYK2^-/-^ host mice, 4 weeks after intrasplenic injection of syngeneic MC38 CRC cells. (**h**) Histomorphometric quantification of the tumor load (% of tumor area to total tissue area) of TYK2^+/+^ and TYK2^-/-^ host mice, 4 weeks after intrasplenic injection of syngeneic MC38 CRC cells. Bar diagrams represent mean values +/- SEM with each data point representing a mouse. Case Viewer, QuPath and Halo software were used for histomorphometry. Statistical analysis was performed using unpaired Student’s t-test. p values are indicated.

CRC cells also metastasize to the lung and the peritoneum. We investigated TYK2 functions in peritoneal CRC metastasis because the peritoneum has a significantly different immune contexture than the liver. AKP organoids were injected into the peritoneum of TYK2^+/+^ and TYK2^-/-^ host mice, which were sacrificed 8 weeks later. However, the peritoneal environment was no suitable soil for the organoids and they did not form metastases. The experiment was repeated with a second independent AKP organoid clone. This AKP clone also readily metastasized to the liver after intrasplenic injection with significantly increased tumor burden in TYK2^-/-^ host mice (Supplementary Figure 2k-n), but failed to form peritoneal metastases. Therefore, syngeneic murine MC38 cells were used, which is an established model for peritoneal CRC metastasis. Interestingly, the peritoneal metastatic burden in this experiment was comparable between TYK2^+/+^ and TYK2^-/-^ host mice (Figure 2a-d), suggesting that TYK2 has a more specific function in hepatic immunosurveillance of metastasis. To support this idea, intrasplenic injections of MC38 cells were performed, which reproduced the data obtained with the organoids and resulted in a significantly increased metastatic burden in TYK2^-/-^ host mice (Figure 2e-h). These experiments demonstrate a key function of TYK2 in immunosurveillance of CRLM.

### The immunophenotype of CRLM is altered in TYK2^-/-^ and TYK2^Δhem^ host mice

**Figure 3:**
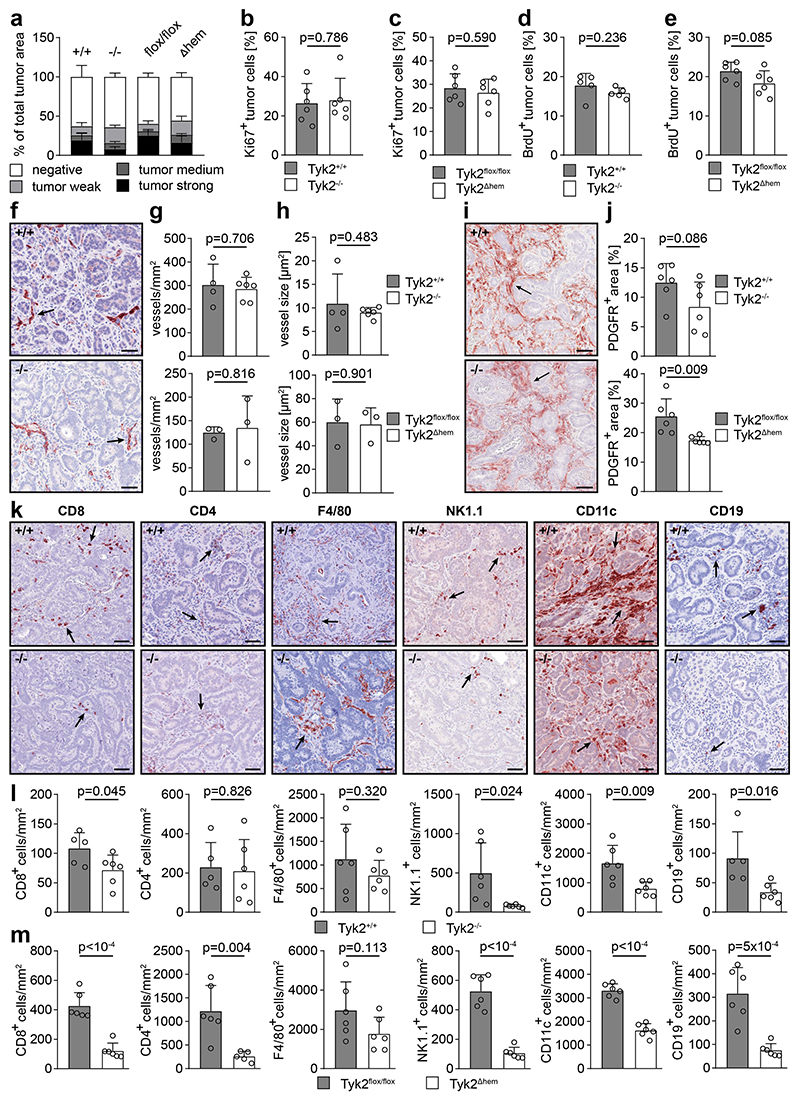
Altered immunophenotype of established liver metastases in TYK2-deficient host mice. (**a**) Histomorphometric quantification of immunohistochemical GFP staining (as shown in Figure 1c, h) in metastases of TYK2^+/+^ (+/+), TYK2^-/-^ (-/-), TYK2^flox/flox^ (flox/flox) and TYK2^Δhem^ (Δhem) host mice. The GFP-negative area (stroma and GFP-negative tumor cells) and GFP-positive area (tumor cells) is given in % of the total tumor area. The parameters of the histomorphometry software were adjusted so that tumor areas with weak (tumor weak), medium (tumor medium) and strong (tumor strong) GFP staining intensity could be separated and quantified. All slides were stained in a row and quantified with the same parameters. (**b**) Histomorphometric quantification of immunohistochemical Ki67 staining of metastases in TYK2^+/+^ and TYK2^-/-^ host mice, calculated as percentage of positive tumor cells per total tumor cells. (**c**) Histomorphometric quantification of immunohistochemical Ki67 staining of metastases in TYK2^flox/flox^ and TYK2^Δhem^ host mice, calculated as percentage of positive tumor cells per total tumor cells. (**d**) Histomorphometric quantification of immunohistochemical BrdU staining of metastases in TYK2^+/+^ and TYK2^-/-^ host mice, calculated as percentage of positive tumor cells per total tumor cells. (**e**) Histomorphometric quantification of immunohistochemical BrdU staining of metastases in TYK2^flox/flox^ and TYK2^Δhem^ host mice, calculated as percentage of positive tumor cells per total tumor cells. (**f**) Immunohistochemical staining for the blood vessel marker endomucin in metastases of TYK2^+/+^ (+/+) and TYK2^-/-^ (-/-) host mice. Vessels are indicated by arrows. Scale bar = 50 μm. (**g**) Histomorphometric quantification of blood vessel density in metastases of TYK2^+/+^ and TYK2^-/-^ host mice (upper bar diagram) as well as TYK2^flox/flox^ and TYK2^Δhem^ host mice (lower bar diagram). (**h**) Histomorphometric quantification of blood vessel size in metastases of TYK2^+/+^ and TYK2^-/-^ host mice (upper bar diagram) as well as TYK2^flox/flox^ and TYK2^Δhem^ host mice (lower bar diagram). (**i**) Immunohistochemical staining for the fibroblast marker PDGFR in metastases of TYK2^+/+^ (+/+) and TYK2^-/-^ (-) host mice. Fibroblasts are indicated by arrows. Scale bar = 50 μm. (**j**) Histomorphometric quantification of PDGFR-positive area in metastases of TYK2^+/+^ and TYK2^-/-^ host mice (upper bar diagram) as well as TYK2^flox/flox^ and TYK2^Δhem^ host mice (lower bar diagram). (**k**) Immunohistochemical staining of CD8, CD4, F4/80, NK1.1, CD11c and CD19 in metastases of TYK2^+/+^ (+/+) and TYK2^-/-^ (-/-) host mice. Positive immune cells are indicated by arrows. Scale bar = 50 μm. (**l**) Histomorphometric quantification of CD8^+^, CD4^+^, F4/80^+^, NK1.1^+^, CD11c^+^ and CD19^+^ immune cells in metastases of TYK2^+/+^ and TYK2^-/-^ host mice. (**m**) Histomorphometric quantification of CD8^+^, CD4^+^, F4/80^+^, NK1.1^+^, CD11c^+^ and CD19^+^ immune cells in metastases of TYK2^flox/flox^ and TYK2^Δhem^ host mice. All analyses were performed with mice, 4 weeks after intrasplenic injection of AKP organoids. Bar diagrams represent mean values +/- SEM with each data point representing a mouse. Case Viewer, QuPath and Halo software were used for histomorphometry. Statistical analysis was performed using unpaired Student’s t-test. p values are indicated.

We characterized tumor parameters and immunophenotypes of liver metastases in TYK2^-/-^ and TYK2^Δhem^ host mice four weeks after intrasplenic injection of AKP organoids. The extent of stromalization was determined by histomorphometry of GFP-stained liver sections (Figure 1c, h). Tumor and stromal tissue were differentiated based on their histological appearance and GFP expression, which was only present in tumor cells. The relative percentage of GFP-expressing metastatic tumor tissue and GFP-negative stroma was similar in TYK2-proficient and TYK2-deficient host mice despite the significant difference in tumor burden (Figure 3a). Interestingly, histomorphometric quantification of GFP staining intensity did not show immune-mediated counter-selection for tumor cells with high GFP expression in TYK2-proficient host mice, as would be expected if GFP acts as a tumor-specific antigen (41). Rather, there was lower GFP expression in tumor cells of TYK2-deficient host mice although the reduction was non-significant (Figure 3a). We also injected a GFP-negative AKP organoid clone into the spleen of TYK2^+/+^ and TYK2^-/-^ host mice. The metastatic potential of this clone was rather low, however, the micrometastatic lesions found in TYK2^-/-^ host mice were significantly larger than in TYK2^+/+^ host mice (Supplementary Figure 3a, b). This suggests that the function of TYK2 in metastatic immunosurveillance does not depend on GFP reporter expression.

Metastatic proliferation, as assessed by immunohistochemistry for Ki67 (Figure 3b, c, Supplementary Figure 3c) and BrdU incorporation (Figure 3d, e, Supplementary Figure 3c), as well as blood vessel density and blood vessel size in metastases, both assessed by immunohistochemistry for endomucin (Figure 3f-h), were similar in TYK2-proficient and TYK2-deficient host mice. Staining for PDGFR showed a tendency toward a lower number of cancer-associated fibroblasts (CAFs) in metastases of TYK2^-/-^ host mice, which was significant in TYK2^Δhem^ host mice (Figure 3i, j).

To quantify the number of CD8^+^ T cells, CD4^+^ T cells, macrophages, NK cells, dendritic cells (DCs) and B cells, immunophenotyping was performed by immunohistochemistry for CD8, CD4, F4/80, NK1.1, CD11c and CD19, respectively (Figure 3k). A significant reduction in CD8^+^ T cells, NK cells, DCs and B cells in the metastases of TYK2^-/-^ host mice was revealed by histomorphometry of stained sections, while the number of CD4^+^ T cells and macrophages was not significantly altered (Figure 3l). This immunophenotype was largely phenocopied in metastases of TYK2^Δhem^ host mice, with the exception of CD4^+^ T cells, which were also significantly reduced (Figure 3m).

The relative proportion of infiltrated immune cell types was analyzed in dissected liver metastases. Flow cytometric analysis revealed a significant reduction in the proportion of T cells to total CD45^+^ cells in metastases of TYK2-deficient host mice, while the proportion of NK cells was not altered (Supplementary Figure 3d-h). In contrast, the relative number of CD11b^+^ myeloid cells was increased (Supplementary Figure 3i, j). A detailed analysis of the myeloid population revealed that this increase was mainly due to a higher proportion of granulocytes, while the proportion of macrophages and DCs did not change (Supplementary Figure 3k-m). Taken together, these data suggest that the immunophenotype of AKP liver metastasis differs between TYK2-proficient and TYK2-deficient host mice, with a marked reduction of adaptive T cell infiltration in TYK2-deficient hosts.

### TYK2 is essential for immunosurveillance of micrometastatic lesions

**Figure 4:**
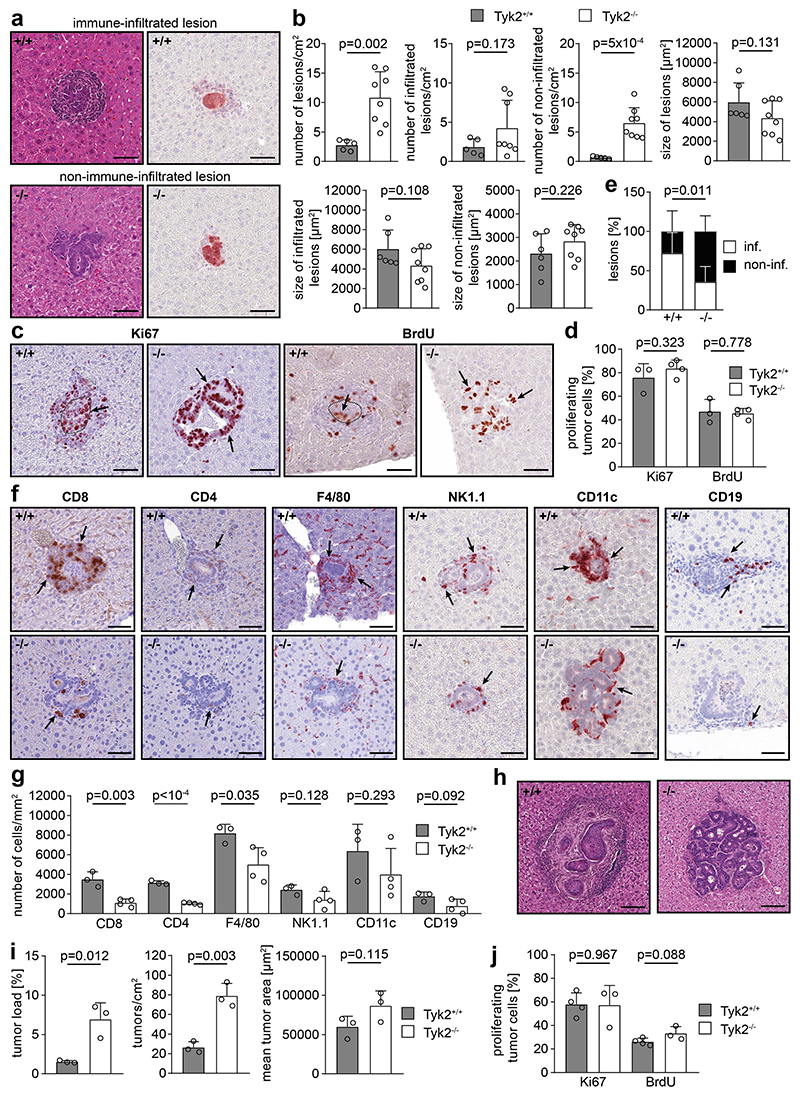
Altered immunophenotype of micrometastatic lesions in TYK2^-/-^ host mice. (**a**) H&E (left images) and GFP staining (right images) of consecutive liver sections of TYK2^+/+^ (+/+) and TYK2^-/-^ (-/-) host mice, 7 days after intrasplenic injection of AKP organoids. Representative metastatic lesions are shown. Tumor cells in GFP-stained images are red. Scale bar = 50 μm. (**b**) Number and size of total metastatic lesions, infiltrated metastatic lesions and non-infiltrated metastatic lesions in TYK2^+/+^ and TYK2^-/-^ host mice, 7 days after intrasplenic injection of AKP organoids. (**c**) Immunohistochemical staining of metastatic lesions for Ki67 (left images) and BrdU (right images) in TYK2^+/+^ (+/+) and TYK2^-/-^ (-/-) host mice, 7 days after intrasplenic injection of AKP organoids. Positive nuclei are indicated by arrows. Scale bar = 50 μm. (**d**) Histomorphometric quantification of proliferating tumor cells in metastatic lesions in TYK2^+/+^ and TYK2^-/-^ host mice, 7 days after intrasplenic injection of AKP organoids, calculated as percentage of Ki67-or BrdU-positive tumor cells per total tumor cells. (**e**) Percentage of immune-infiltrated (inf.) versus non-immune-infiltrated (non-inf.) lesions in TYK2^+/+^ and TYK2^-/-^ host mice, 7 days after intrasplenic injection of AKP organoids. (**f**) Immunohistochemical staining of CD8, CD4, F4/80, NK1.1, CD11c and CD19 in micrometastatic lesions of TYK2^+/+^ (+/+) and TYK2^-/-^ (-/-) host mice, 7 days after intrasplenic injection of AKP organoids. Positive immune cells are indicated by arrows. Scale bar = 50 μm. (**g**) Histomorphometric quantification of CD8^+^, CD4^+^, F4/80^+^, NK1.1^+^, CD11c^+^ and CD19^+^ immune cells in metastases of TYK2^+/+^ and TYK2^-/-^ host mice, 7 days after intrasplenic injection of AKP organoids. (**h**) H&E staining of metastases in TYK2^+/+^ (+/+) and TYK2^-/-^ (-/-) host mice, 14 days after intrasplenic injection of AKP organoids. Representative metastatic lesions are shown. Scale bar = 100 μm. (**i**) Tumor load (left bar diagram, % of tumor area to total tissue area), tumor number (middle bar diagram) and tumor size (right bar diagram) of metastasis in TYK2^+/+^ and TYK2^-/-^ host mice 14 days after intrasplenic injection of AKP organoids. (**j**) Histomorphometric quantification of immunohistochemical Ki67 and BrdU staining of tumors in TYK2^+/+^ and TYK2^-/-^ host mice, 14 days after intrasplenic injection of AKP organoids, calculated as percentage of positive tumor cells per total tumor cells. Bar diagrams represent mean values +/- SEM with each data point representing a mouse. Case Viewer, QuPath and Halo software were used for histomorphometry. Statistical analysis was performed using unpaired Student’s t-test. p values are indicated.

We observed an inverse correlation of metastatic burden with infiltration of different immune cell types into metastases (Supplementary Figure 4a-f). This raised the question whether the reduced immune infiltration in metastases of TYK2-deficient host mice is directly due to TYK2 deficiency or is an indirect consequence of increased tumor burden. Therefore, immunophenotyping was performed at earlier stages of metastasis. Micrometastatic lesions, consisting of few GFP-positive tumor cells, could be detected 7 days after intrasplenic injection of AKP organoids (Figure 4a). TYK2^-/-^ host mice contained a higher number of micrometastases compared to TYK2^+/+^ host mice already at this early metastatic stage (Figure 4b). A tendency toward a higher number of micrometastases was observed in TYK2^Δhem^ host mice (Supplementary Figure 5a). However, the size of micrometastases was comparable in TYK2-deficient and TYK2-proficient host mice (Figure 4b, Supplementary Figure 5a). Consistently, no difference in tumor cell proliferation between metastases in TYK2^+/+^ and TYK2^-/-^ host mice was revealed by quantification of Ki67 and BrdU staining (Figure 4c, d). However, differential immune infiltration of these micrometastases was observed. While most micrometastases of TYK2-proficient host mice were strongly immune-infiltrated and surrounded by a ring-shaped immune structure, a considerable proportion of lesions of TYK2-deficient host mice were only weakly infiltrated (Figure 4a, b, e, Supplementary Figure 5a, b). The total number of immune-infiltrated lesions was comparable between genotypes, while the total number non-infiltrated lesions was increased in TYK2-deficient mice (Figure 4b, Supplementary Figure 5a). Immunohistochemical staining of major immune cell populations revealed a significant reduction of CD8^+^ and CD4^+^ T cell and macrophages and a tendency towards a reduction of NK cells, DCs and B cells in TYK2^-/-^ host mice (Figure 4f, g).

Metastases in TYK2^+/+^ and TYK2^-/-^ host mice showed different histological morphologies 14 days after intrasplenic injection of AKP organoids. While lesions in TYK2^-/-^ hosts thrived, several lesions in TYK2^+/+^ hosts appeared to be immunologically obliterated, leaving a scar-like structure behind (Figure 4h). In addition, metastatic burden and multiplicity were significantly increased at 14 days in TYK2^-/-^ host mice, while a tendency towards an increase in these parameters was observed in TYK2^Δhem^ host mice (Figure 4i, Supplementary Figure 5c). Similar to 7 days, the size of the metastatic lesions and proliferation was not altered at 14 days in TYK2-deficient host mice (Figure 4i, j, Supplementary Figure 5c). Immunological characterization showed decreased immune infiltration with significantly lower numbers of CD8^+^ T cells and B cells as well as a tendency towards a reduced number of CD4^+^ T cells, macrophages, NK cells and DCs in metastases of TYK2^-/-^ host mice (Supplementary Figure 5e). Taken together, these data suggest that TYK2 is required for immunosurveillance of early metastatic lesions.

### Immunosurveillance of CRLM is mediated by TYK2 signaling in DCs

**Figure 5:**
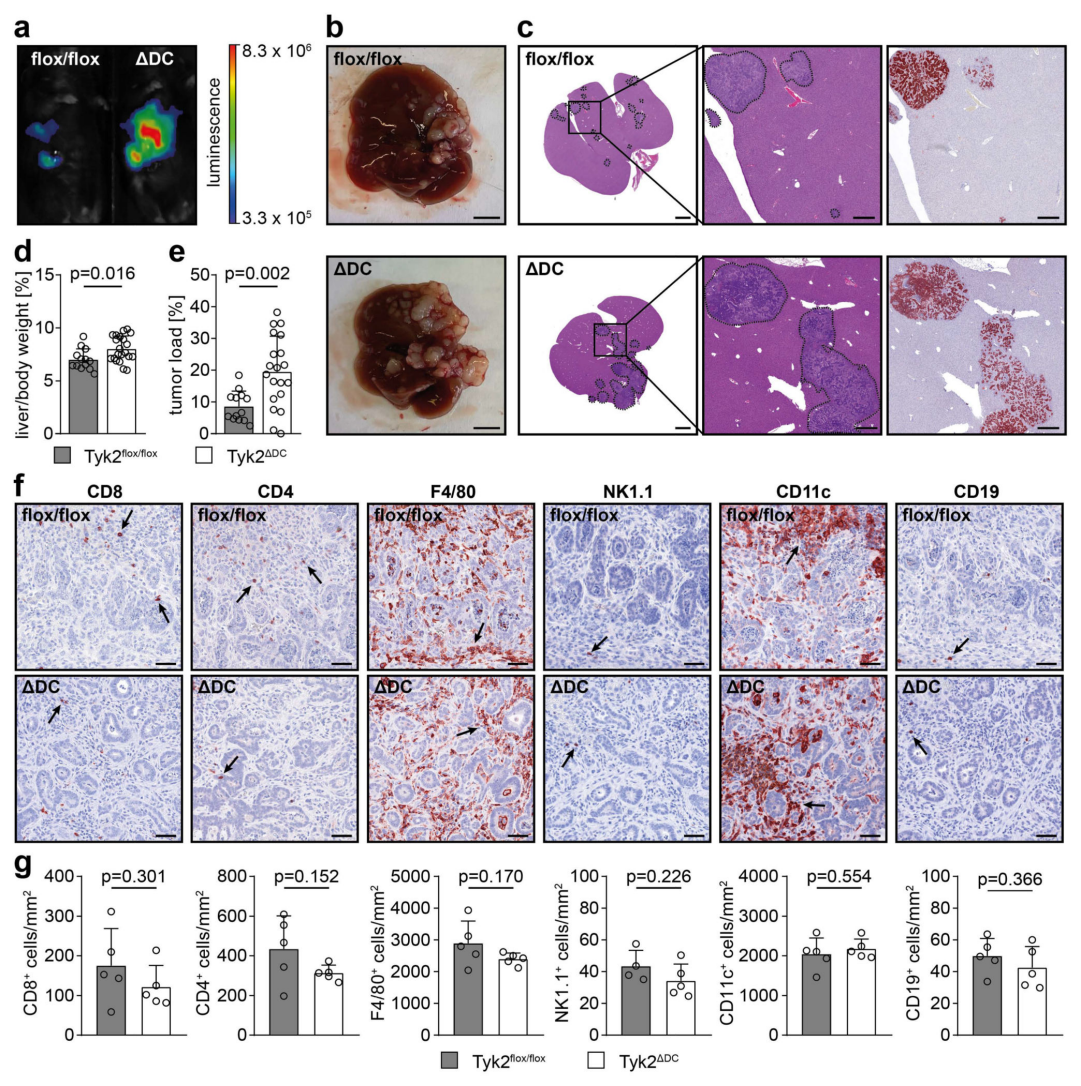
TYK2 in dendritic cells is required for immunosurveillance of CRLM. (**a**) Representative IVIS images of TYK2^flox/flox^ (flox/flox) and TYK2^ΔDC^ (ΔDC) host mice, 3 weeks after intrasplenic injection of AKP organoids. (**b**) Macroscopic images of livers of TYK2^flox/flox^ (flox/flox) and TYK2^ΔDC^ (ΔDC) host mice, 4 weeks after intrasplenic injection of AKP organoids. Scale bar = 5 mm. (**c**) H&E and GFP staining of liver sections of TYK2^flox/flox^ (flox/flox, upper images) and TYK2^ΔDC^ (ΔDC, bottom images) host mice, 4 weeks after intrasplenic injection of AKP organoids. The images in the center represent higher magnifications of the images on the left, with the square indicating the magnified region. Dashed lines mark metastatic lesions used to quantify the tumor load shown in (e). The images on the right show immunohistochemical GFP staining of consecutive sections. Tumor cells are red. Scale bar = 2 mm for the images on the left and 500 μm for the images in the center and on the right. (**d**) Liver-to-body weight ratio of TYK2^flox/flox^ and TYK2^ΔDC^ host mice, 4 weeks after intrasplenic injection of AKP organoids. (**e**) Histomorphometric quantification of the tumor load (% of tumor area to total tissue area) of TYK2^flox/flox^ and TYK2^ΔDC^ host mice, 4 weeks after intrasplenic injection of AKP organoids. (**f**) Immunohistochemical staining of CD8, CD4, F4/80, NK1.1, CD11c and CD19 in metastases of TYK2^flox/flox^ (flox/flox) and TYK2^ΔDC^ (ΔDC) host mice, 4 weeks after intrasplenic injection of AKP organoids. Positive immune cells are indicated by arrows. Scale bar = 50 μm. (**g**) Histomorphometric quantification of CD8^+^, CD4^+^, F4/80^+^, NK1.1^+^, CD11c^+^ and CD19^+^ immune cells in metastases of TYK2^flox/flox^ and TYK2^ΔDC^ host mice, 4 weeks after intrasplenic injection of AKP organoids. Bar diagrams represent mean values +/- SEM with each data point representing a mouse. Case Viewer, QuPath and Halo software were used for histomorphometry. Statistical analysis was performed using unpaired Student’s t-test. p values are indicated.

To functionally characterize immune cell types that require TYK2 for an efficient anti-metastatic immune response, several conditional mouse models were used. We focused on the immune cell types that were altered in AKP metastases of TYK2^-/-^ and TYK2^Δhem^ host mice. Therefore, AKP organoids were injected into the spleen of TYK2^ΔM^ (LysM-cre TYK2^flox/flox^), TYK2^ΔT^ (CD4-cre TYK2^flox/flox^), TYK2^ΔNK^ (Ncr1-cre TYK2^flox/flox^) and TYK2^ΔDC^ (CD11c-cre TYK2^flox/flox^) host mice, which have a specific deletion of *Tyk2* in myeloid cells, T cells, NK cells and DCs, respectively. The deletion in the respective immune cell types was confirmed by *in vitro* differentiation assays and purification of immune cell populations by cell sorting (Supplementary Figure 6a-l). LysM-cre induces deletion of floxed alleles in macrophages (83-98%) and granulocytes (near 100%), which include neutrophils, whereas deletion in DCs is inefficient (42). Notably, successful deletion of *Tyk2* could also be detected in purified Kupffer cells of TYK2^ΔM^ mice (Supplementary Figure 6c, d). Corresponding TYK2^ΔM^ host mice did not show increased metastatic burden after intrasplenic AKP organoid injection, suggesting that TYK2 signaling in macrophages, Kupffer cells and granulocytes is dispensable for immunosurveillance of CRLM (Supplementary Figure 7a-e). Similarly, TYK2^ΔT^ (Supplementary Figure 7f-j) and TYK2^ΔNK^ host mice (Supplementary Figure 7k-o) did not show increased metastasis. In contrast, TYK2^ΔDC^ host mice showed a significant increase in metastatic burden (Figure 5a-e) indicating an important function of dendritic TYK2 in immunosurveillance of CRLM. However, TYK2^ΔDC^ mice did not phenocopy the pronounced degree of reduction of immune infiltration in metastatic lesions observed in TYK2^-/-^ and TYK2^Δhem^ host mice, and no significant reduction in T cells, macrophages, NK cells and B cells was found (Figure 5f, g).

**Figure 6:**
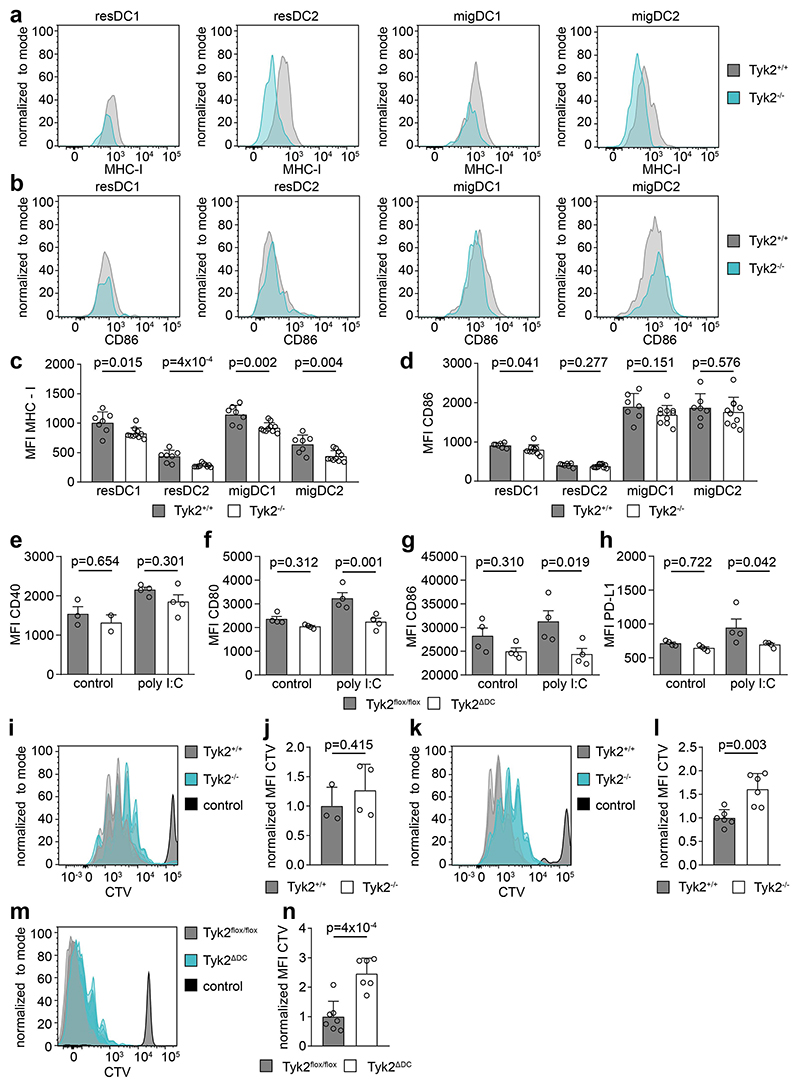
Reduced MHC-I expression and impaired antigen cross presentation of TYK2-deficient DCs. (**a**) Representative flow cytometry plots for the mean fluorescence intensity (MFI) of MHC-I on resident (res) and migratory (mig) cDC1 and cDC2 populations, isolated from the lymph nodes of metastases-bearing mice. (**b**) Representative flow cytometry plots for the MFI of CD86 on resident (res) and migratory (mig) DC1 and DC2 populations, isolated from the lymph nodes of metastases-bearing mice. (**c**) Bar diagram for the MFI of MHC-I on resident (res) and migratory (mig) DC1 and DC2 populations, isolated from the lymph nodes of metastases-bearing mice. (**d**) Bar diagram for the MFI of CD86 on resident (res) and migratory (mig) DC1 and DC2 populations, isolated from the lymph nodes of metastases-bearing mice. (**e-h**) Bar diagrams for the MFI of CD40 (e), CD80 (f) CD86 (g) and PD-L1 (h) on FACS-purified DCs, ex vivo unstimulated (control) or stimulated with poly I:C. The DCs were isolated from the spleen of TYK2^flox/flox^ and TYK2^ΔDC^ mice. (**i**) Flow cytometry plot indicating the proliferation of adoptively transferred, CTV-labeled OT-II T cells into TYK2^+/+^ and TYK2^-/-^ mice after stimulation with ovalbumin. Dilution of the label indicates proliferation. (**j**) Quantification of OT-II T cell proliferation via MFI of CTV (normalized to control) in TYK2^+/+^ and TYK2^-/-^ mice. (**k**) Flow cytometry plot indicating the proliferation of adoptively transferred, CTV-labeled OT-I T cells into TYK2^+/+^ and TYK2^-/-^ mice after stimulation with ovalbumin. Dilution of the label indicates proliferation. (**l**) Quantification of OT-I T cell proliferation via MFI of CTV (normalized to control) in TYK2^+/+^ and TYK2^-/-^ mice. (**m**) Flow cytometry plot indicating the proliferation of adoptively transferred, CTV-labeled OT-I T cells into TYK2^flox/flox^ and TYK2^ΔDC^ mice after stimulation with ovalbumin. Dilution of the label indicates proliferation. (**n**) Quantification of OT-I T cell proliferation via MFI of CTV (normalized to control) in TYK2^flox/flox^ and TYK2^ΔDC^ mice. Bar diagrams represent mean values +/- SEM with each data point representing a mouse. Flowjo software was used to analyze flow cytometry data. Statistical analysis was performed using unpaired Student’s t-test. p values are indicated.

Cross presentation of antigens via MHC-I to CD8^+^ T cells is an important function of DCs in cancer immunosurveillance (43). We therefore examined surface expression of MHC-I and the co-activating molecule CD86 on resident and migratory cDCs in the tumor-draining lymph nodes of metastasis-bearing mice (Figure 6a-d, Supplementary Figure 8a-c). While the frequency of resident and migratory cDC1 and cDC2 immune cells was not changed in the tumor-draining lymph nodes of TYK2^-/-^ host mice (Supplementary Figure 8a-c), surface expression of MHC-I was reduced in all four cDC subpopulations (Figure 6a, c). In contrast, expression of the co-activating molecule CD86 was mostly unchanged (Figure 6b, d). However, a significant reduction of CD80, CD86 and PD-L1 protein expression was found ex vivo in TYK2-deficient FACS-sorted DCs stimulated with poly I:C (Figure 6e-h, Supplementary Figure 8d, e). Bulk RNA sequencing was performed using RNA from healthy livers without metastases, isolated metastases and liver tissue adjacent to metastases to obtain further in vivo information on the functions of TYK2 in immunosurveillance. Markers for immune cell identity were downregulated in metastases from TYK2^Δhem^ host mice which is consistent with the reduced immune infiltration (Supplementary Figure 9). Expression of angiogenic markers produced by myeloid and other immune cells remained largely unchanged, but *Vegfa* expression was significantly upregulated (Supplementary Figure 9). Expression of *type I IFN* could not be detected in the RNA sequencing data but *Ifn-γ* expression was significantly reduced in metastases from TYK2^Δhem^ host mice (Supplementary Figure 10a, c). We also observed downregulation of the T cell-attracting chemokines *Cxcl9* and *Cxcl10*, whose expression is regulated by IFN-γ (Supplementary Figure 10a). The effects of *Tyk2* deletion on other cytokines were less pronounced (Supplementary Figure 10a). Several costimulatory molecules and immune checkpoints were also downregulated in metastases from TYK2^Δhem^ host mice (Supplementary Figure 10b, c). Downregulation of some immune checkpoints was unexpected as they are T cell exhaustion markers. However, downregulation could also result from reduced immune infiltration. To address this issue, T cells were double-stained for CD8 and the exhaustion marker PD-1. Almost all CD8-positive T cells were also positive for PD-1 but the staining is not quantitative (Supplementary Figure 11). Therefore, we injected CTV-labeled CD8^+^ OT-I and CD4^+^ OT-II antigen-specific T cells into TYK2^+/+^ and TYK2^-/-^ mice, followed by intraperitoneal injection of ovalbumin, to further examine the ability of TYK2-deficient DCs to stimulate T cell proliferation. Interestingly, while the proliferation of CD4^+^ OT-II T cells was not affected (Figure 6i, j), CD8^+^ OT-I T cells proliferated significantly less in TYK2^-/-^ mice compared to TYK2^+/+^ mice (Figure 6k, l). Adoptive transfer of labeled CD8^+^ OT-I T cell into TYK2^ΔDC^ mice confirmed a cell-intrinsic defect of DCs in stimulating CD8^+^ T cell proliferation (Figure 6m, n). Therefore, TYK2-deficient DCs have a lower potential to trigger an effective antigen-specific cytotoxic T cell response in CRLM.

### TYK2 is mainly expressed in human CRLM-enriched LAMP3^+^ CCR7^+^ conventional dendritic cells

**Figure 7:**
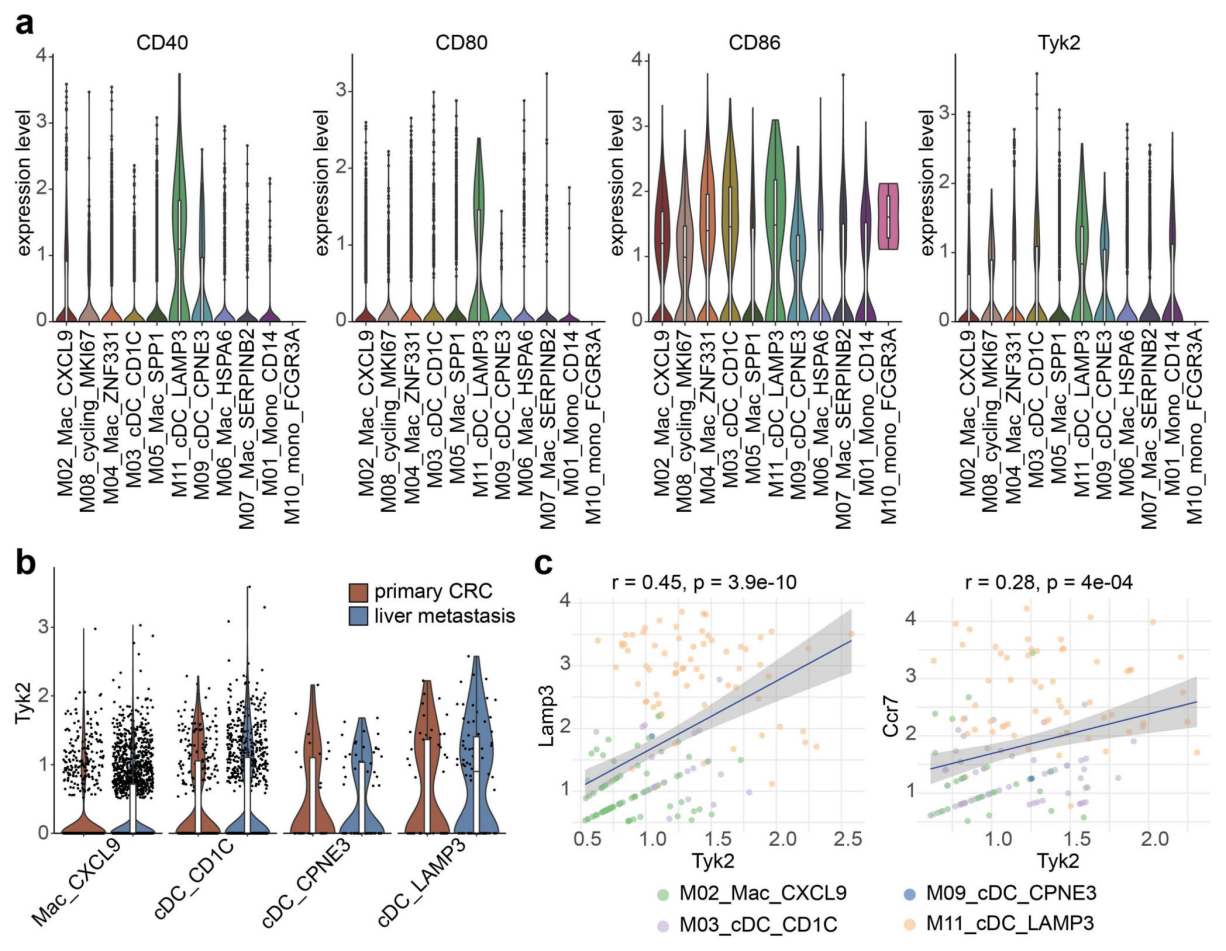
Correlation of mRNA expression of *TYK2* and *LAMP3* as well as *TYK2* and *CCR7* in human CRC-derived cDCs. (**a**) Violin plots for expression of activation markers *CD40, CD80, CD86* and *TYK2* in myeloid cells from primary CRC and CRLM using a published scRNA sequencing dataset. (**b**) Violin plot for *TYK2* expression in dendritic cell populations and CXCL9-expressing macrophages in primary CRC and CRLM. (**c**) Correlation plot for mRNA expression of *TYK2* with *LAMP3* as well as *TYK2* with *CCR7* in dendritic cell populations and CXCL9-expressing macrophages.

Kaplan-Meier plotter analysis revealed that patients with high *TYK2* expression in CRC had worse overall survival (Supplementary Figure 12a). However, the difference between the patient cohorts was not very pronounced and analysis of bulk mRNA expression does not distinguish between *TYK2* in tumor cells and stromal cells. Therefore, we analyzed a scRNA-seq dataset with 41 immune cell clusters in primary CRC, CRLM and corresponding adjacent tissues (36). The dataset contained three clusters of conventional dendritic cells (cDCs): cDC_CD1c (corresponding to cDC2), cDC_CPNE3 (corresponding to cDC1) and a CRLM-enriched cDC_LAMP3 population (36). cDC_LAMP3 expressed high levels of the chemokine receptor *CCR7*, suggesting that they are predestined for homing to tumor-draining lymph nodes (36). Furthermore, they showed high expression of the costimulatory molecules *CD40, CD80* and *CD86* (36). Our analyses demonstrated that *TYK2* is predominantly expressed in cDC_LAMP3, a distinct population of activated cDCs in tumors (Supplementary Figure 12b, c). We focused our analyses on the cDC clusters and included also the Mac_CXCL9 macrophage cluster that is enriched for the IFN-γ response and T cell activation pathways(36). Our analyses confirmed high expression of costimulatory molecules (Figure 7a) and CRLM enrichment (Figure 7b) of the cDC_LAMP3 population. Furthermore, correlation analysis revealed a positive correlation between *LAMP3/TYK2* as well as *CCR7/TYK2* in the clusters (Figure 7c). In summary, these data demonstrate that *TYK2* is predominantly expressed in human LAMP3^+^ CCR7^+^ cDCs that are enriched in CRLM and predicted to activate T cells in tumor-draining lymph nodes.

### Pharmacological inhibition of TYK2 promotes CRLM

**Figure 8:**
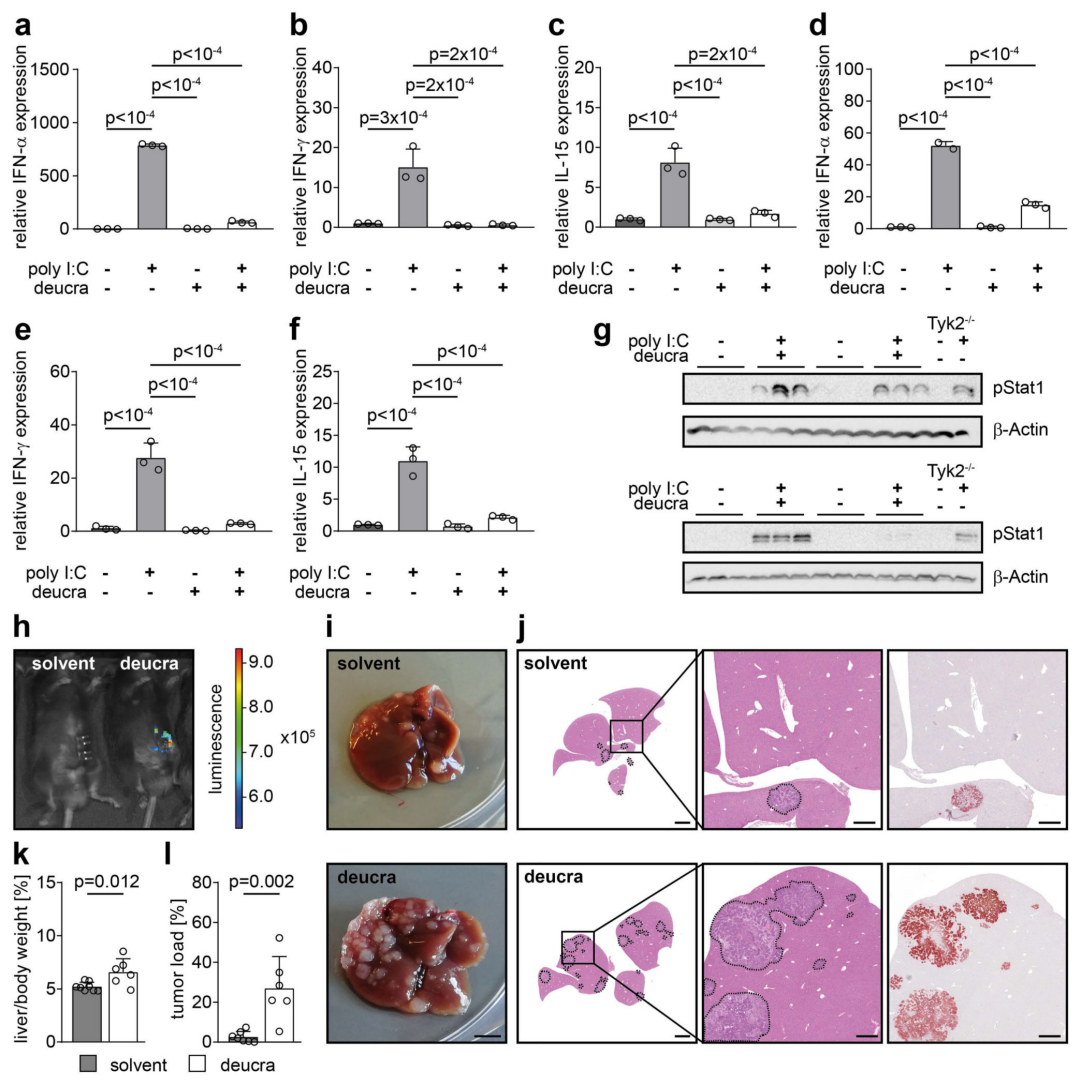
Pharmacological inhibition of TYK2 promotes liver metastasis of AKP organoids. (**a-f**) qPCR analysis for *Ifn-α* (a, d), *Ifn-γ* (b, e) and *IL-15* (c, f) mRNA expression in isolated splenocytes (a-c) and liver tissue (d-f) from C57BL/6 mice, treated with poly I:C and/or deucravacitinib (deucra). Expression values were normalized to untreated controls and *Gapdh*. (**g**) Western blot for activated (Tyr-701-phosphorylated) pSTAT1 protein in isolated splenocytes (upper two blots) and liver tissue (lower two blots) of C57BL/6 mice, treated with poly I:C and deucravacitinib. Splenocytes or liver tissue form TYK2^-/-^ mice were included as control. β-Actin was used as loading control. (**h**) Representative IVIS images of C57BL/6 host mice, treated with solvent or the TYK2 inhibitor deucravacitinib (deucra), 3 weeks after intrasplenic injection of AKP organoids. (**i**) Macroscopic images of livers of C57BL/6 host mice, treated with solvent or deucravacitinib, 4 weeks after intrasplenic injection of AKP organoids. Scale bar = 5 mm. (**j**) H&E and GFP staining of liver sections of C57BL/6 host mice, treated with solvent (upper images) or deucravacitinib (bottom images), 4 weeks after intrasplenic injection of AKP organoids. The images in the center represent higher magnifications of the images on the left, with the square indicating the magnified region. Dashed lines mark metastatic lesions used to quantify the tumor load shown in (l). The images on the right show immunohistochemical GFP staining of consecutive sections. Tumor cells are red. Scale bar = 2 mm for the images on the left and 500 μm for the images in the center and on the right. (**k**) Liver-to-body weight ratio of C57BL/6 host mice, treated with solvent or deucravacitinib, 4 weeks after intrasplenic injection of AKP organoids. (**l**) Histomorphometric quantification of the tumor load (% of tumor area to total tissue area) of C57BL/6 host mice, treated with solvent or deucravacitinib, 4 weeks after intrasplenic injection of AKP organoids. Bar diagrams represent mean values +/- SEM with each data point representing a mouse. Case Viewer, QuPath and Halo software were used for histomorphometry. Statistical analysis was performed using ANOVA (a-f) or unpaired Student’s t-test (k, l). p values are indicated.

The novel TYK2 inhibitor deucravacitinib was recently approved by the FDA to treat patients with plaque psoriasis and is currently being tested in clinical trials for the treatment of other inflammatory disorders (21). Therefore, we investigated whether pharmacological TYK2 inhibition can promote CRLM to a similar extend as genetic TYK2 deletion. The efficacy of TYK2 inhibition by deucravacitinib was tested in mice treated with the interferon inducer Poly I:C. This double-stranded RNA induced expression of *Ifn-α, Ifn-γ* and *IL-15* as well as Tyr-701 phosphorylation of STAT1, as demonstrated by qPCR and Western blot analysis. Oral administration of deucravacitinib significantly attenuated these responses, confirming successful inhibition of TYK2 (Figure 8a-g). We injected AKP organoids into the spleen of C57BL/6 host mice that were pre-treated with deucravacitinib one day prior to injection. The inhibitor was administered orally every day for four weeks in order to efficiently block TYK2 for the entire duration of the experiment. The metastatic burden in the liver of host mice with TYK2 inhibition was significantly increased compared to control hosts (Figure 8h-l). These results suggest that pharmacological inhibition of TYK2 promotes CRLM.

## Discussion

Several studies have shown that TYK2 is a cancer cell-intrinsic oncogenic driver in hematopoietic malignancies (5,6,33,44–49). However, there are very limited data on its cancer cell-intrinsic functions in solid tumors, especially metastases. Recently, we have shown that the burden of AOM-DSS-induced autochthonous primary CRC is increased in mice with conditional deletion of *Tyk2* in intestinal epithelial cells (12), but these chemically-induced tumors do not metastasize. To investigate the cancer cell-intrinsic functions of TYK2 in CRLM, we injected CRC organoids with CRISPR/Cas9-mediated *Tyk2* deletion into the spleen. Intrasplenic injection of CRC cells enables efficient hepatic seeding and models colonization, which is the most inefficient step in metastasis and a major focus of our study. Alternative experimental strategies, such as intracecal or intramucosal injection of CRC cells (50,51), model earlier steps of the invasion-metastasis cascade but hepatic seeding tends to be inefficient. Instead of CRC cell lines, we used organoids derived from spontaneously developing polyps of mice carrying mutations in genes critical for human CRC development (26). The 3D propagation of organoids preserves their stem-like properties, which are very similar to metastatic cells from primary tumor (52). Immunosurveillance and immune evasion of organoid-derived metastases may therefore closely resemble naturally occurring immune responses. We found that deletion of *Tyk2* in the organoids had no significant effect on CRLM. These data suggest that cancer cell-intrinsic TYK2 signaling is not required for metastatic colonization of the liver. However, it is still possible that TYK2 has cancer cell-intrinsic roles at earlier stages of the CRLM invasion-metastasis cascade.

TYK2 is activated in immune cells by several cytokines including type I IFNs, resulting in a feed-forward loop with production of additional cytokines. Blunting of this response affects cancer immunosurveillance of several innate and adaptive immune cell types (13,53). According to the current view, IFN-α and IFN-β stimulate the production of IL-12, IL-15, IL-23 and IFN-γ by myeloid cells in a TYK2-dependent manner. These cytokines bind to TYK2-dependent receptors on CTLs and NK cells, provoking further IFN-γ production and immune activation (13,53). However, it is not clear whether TYK2 is required for immunosurveillance of CRLM and if so, in which cell type. We observed a significantly increased metastatic liver colonization in TYK2^-/-^ and TYK2^Δhem^ host mice, as well as in wild-type mice treated with deucravacitinib, suggesting a key function in immunosurveillance of CRLM. Interestingly, the number of peritoneal CRC metastases was not increased in TYK2^-/-^ host mice, suggesting that TYK2 does not generally promote metastatic immune surveillance. Liver metastases from TYK2-deficient host mice showed significantly reduced immune infiltration, especially of adaptive T cells. The inefficient immune infiltration was already evident in micrometastatic lesions of TYK2-deficient host mice, which rarely showed the ring-shaped immune structure that surrounded most micrometastases of wild-type host mice. The tumor data in host mice with deletion of *Tyk2* in specific immune subsets showed that TYK2 signaling in T cells, NK cells, macrophages, neutrophils or Kupffer cells is not required for immunosurveillance of CRLM. NK cells and Kupffer cells were described as key immune cells for the initial eradication of metastatic CRC cells stuck in the hepatic sinusoids (18). It is likely that these innate immune cells also kill many AKP organoids in our colonization model, but this appears to be TYK2-independent. Increased metastatic burden was only observed in TYK2^ΔDC^ host mice, identifying CD11c^+^ DCs as a key component of the anti-metastatic immune response. Interestingly, subcutaneous injection of murine CRC cancer cells resulted in an increased tumor burden in TYK2^-/-^ but not in TYK2^ΔDC^ host mice (25). This indicates that TYK2 also mediates immunosurveillance of primary transplanted colorectal tumors but, unlike CRLM, is not specifically required in DCs.

TCGA data showed improved survival in patients with low *TYK2* expression, which is contrary to our expectations, but bulk RNA data do not distinguish between *TYK2* expression in tumor cells and stromal cells. It is possible that TYK2 upregulation in human tumor cells is oncogenic (54), which could explain this result. The available scRNA sequencing datasets for CRC do not allow for a prognostic study with specific *TYK2* expression in DCs. However, using such as dataset (36), we could show that *TYK2* is prominently expressed in a conventional dendritic cell population positive for LAMP3 and CCR7, the chemokine receptor for homing to tumor-draining lymph nodes. Furthermore, the population expressed high levels of T cell costimulatory molecules and was enriched in CRC liver metastases. It is possible that this cDC population has an important TYK2-dependent function in immunosurveillance of CRLM in human patients.

Functional assays revealed that TYK2-deficient DCs exhibit reduced expression of MHC-I and a corresponding dysfunctional cross-presentation to CD8^+^ T cells, which is most likely responsible for reduced immunosurveillance of CRLM in TYK2-deficient host mice. This result is consistent with previous findings showing a decreased potential of TYK2-deficient DCs in MHC-I-linked antigen presentation during Listeria monocytogenes infection (55) and decreased MHC-I expression in TYK2-deficient DCs, associated with the development of type 1 diabetes (56).

Decreased stimulation of adoptively transferred CD8^+^ OT-I T cells was also observed in TYK2^ΔDC^ host mice, indicating that this defect is DC-intrinsic. However, the increase in metastatic burden was more pronounced in TYK2^-/-^ and TYK2^Δhem^ host mice than in TYK2^ΔDC^ host mice. This is likely due to the additional effect of lower immune infiltration in metastases from TYK2^-/-^ and TYK2^Δhem^ host mice, which was not observed in TYK2^ΔDC^ host mice. Therefore, immune infiltration into the metastases may depend on TYK2 signaling in immune cells other than DCs, although DCs in the TME can produce chemoattractants such as CXCL9 and CXCL10. Of note, our RNA sequencing data showed that the expression of these IFN-γ-induced chemokines was substantially reduced in metastasis of TYK2^Δhem^ host mice. Furthermore, ex vivo stimulation experiments with poly I:C showed reduced induction of the costimulatory molecules CD80 and CD86 in DCs, which likely contributes to reduced immunosurveillance.

We could not detect mRNA expression of *Ifn-α* and *Ifn-β* by RNA sequencing in liver metastases, but strong *Ifn-γ* expression was found in metastases from TYK2^flox/flox^ host mice, which was significantly reduced in metastases from TYK2^Δhem^ host mice. The effects of TYK2 deletion on cytokines that promote IFN-γ expression, such as IL-12, were less pronounced. We also could not detect IL-12 and IL-15 by ELISA in supernatants of ex vivo stimulated DCs and supernatants of healthy livers and liver metastases gave inconclusive results, regardless of the genotype. Therefore, it remains to be shown which cytokine is upstream of TYK2 in the immunosurveillance of CRLM and stimulates IFN-γ production. In summary, or data suggest that TYK2 promotes immunosurveillance of CRLM via antigen cross-presentation to CD8^+^ T cells and induction of co-stimulatory molecules CD80 and CD86. It also promotes IFN-γ production, that seems to be the key cytokine in TYK2-mediated immunosurveillance. IFN-γ may induce CXCL9 and CXCL10 chemokines for further immune cell attraction. However, the orchestration of these events by other cytokines remains to be determined.

JAK inhibitors were approved several years ago for the treatment of autoimmune and inflammatory diseases (57,58). Initially developed inhibitors such as Tofacitinib were used for treatment of rheumatoid arthritis and colitis ulcerosa. They had low specificity for JAKs because they compete with the highly conserved active site of Janus kinases and mainly blocked JAK1, JAK2 and/or JAK3. Meanwhile, a black box warning has been released by the FDA for these inhibitors because of potential heart- and cancer-related side effects (59). Moreover, patients treated with these inhibitors often experience recurrent infections (60). In contrast, the novel TYK2 inhibitor deucravacitinib binds to the allosteric site of TYK2, resulting in highly specific inhibition (21,22). Deucravacitinib was approved in 2022 for the treatment of plaque psoriasis and is currently being tested in clinical trials for various inflammatory diseases including IBD. However, the effects of TYK2 inhibition on cancer development remain unclear. This is a critical issue, as several studies have suggested an association between TYK2-inactivating germline mutations and an increased susceptibility to the development of various types of cancer in humans (16,17,61,62). Our data indicate that TYK2 inhibition by deucravacitinib may promote CRLM. This finding should be carefully considered, especially in IBD patients who are already at increased risk of developing occult primary colorectal cancer. However, we used metastatic models without underlying inflammatory disease and it remains to be shown whether inflammation modulates the function of TYK2 in immunosurveillance of CRLM.

## Supplementary Material

Supplementary Material

## Data Availability

Bulk RNA sequencing raw data generated in this study have been deposited at the Gene Expression Omnibus under the accession number GSE299638. Publicly available data analyzed in this study were obtained from GEO (GSE225857) and the Kaplan-Meier Plotter (https://kmplot.com/analysis/). All other raw data are available upon request from the corresponding author.

## Significance

TYK2 restricts the metastasis of colorectal tumors to the liver by supporting dendritic cell-dependent induction of anti-tumor CD8^+^ T cells, which could impact the use of TYK2 inhibitors in patients.
