# Supplementary material for: TYK2 Promotes Immunosurveillance of Colorectal Cancer Liver Metastasis
Source: Cancer Res. Author manuscript; Available in PMC 2025 Oct 22. (PMC7618269; doi:10.1158/0008-5472.CAN-24-4224)
Supplement: Supplementary Material [file EMS209323-supplement-Supplementary_Material.zip › supp_info_11.pdf]

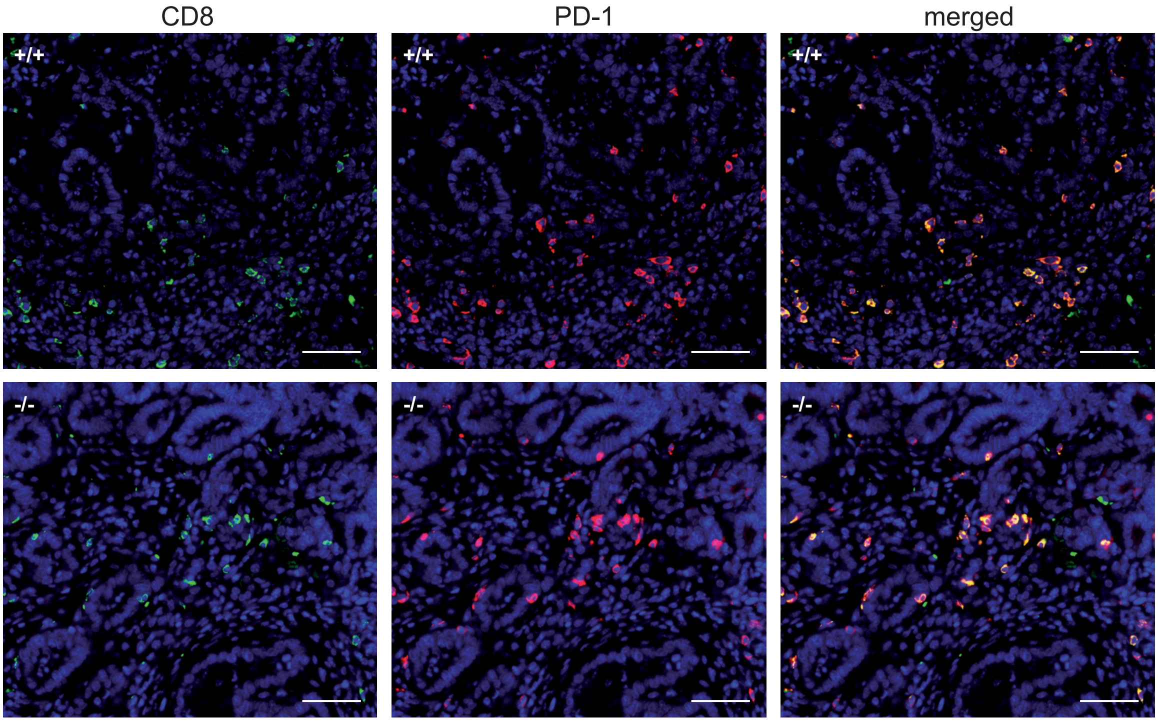

Supplementary Figure 11: Immunofluorescence staining of CD8 (green) and PD-1 (red) in metastases. Liver sections of $TYK2^{flox/flox}$ (flox/flox, upper images) and $TYK2^{\Delta hem}$ (Δhem, bottom images) host mice, 3 weeks after intrasplenic injection of AKP organoids were used. The images show metastatic tissue. Scale bar = 50μm.
