# Supplementary material for: TYK2 Promotes Immunosurveillance of Colorectal Cancer Liver Metastasis
Source: Cancer Res. Author manuscript; Available in PMC 2025 Oct 22. (PMC7618269; doi:10.1158/0008-5472.CAN-24-4224)
Supplement: Supplementary Material [file EMS209323-supplement-Supplementary_Material.zip › supp_info_9.pdf]

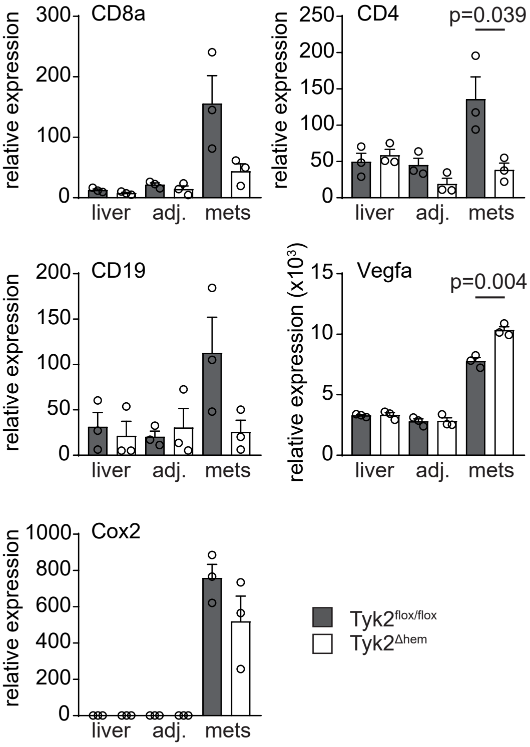

Supplementary Figure 9: mRNA expression of markers for immune cell identity and angiogenic factors in healthy and metastases-bearing livers of TYK2$^{flox/flox}$ and TYK2$^{\Delta hem}$ host mice. Relative expression values were derived from bulk RNA sequencing data. liver: healthy liver without metastases, adj.: liver tissue adjacent to metastatic lesions, mets: tissue from isolated metastases. Bar diagrams represent mean values +/ - SEM with each data point representing a mouse. Statistical analysis was performed using unpaired Student's t-test. p values are indicated.
