# Supplementary material for: TYK2 Promotes Immunosurveillance of Colorectal Cancer Liver Metastasis
Source: Cancer Res. Author manuscript; Available in PMC 2025 Oct 22. (PMC7618269; doi:10.1158/0008-5472.CAN-24-4224)
Supplement: Supplementary Material [file EMS209323-supplement-Supplementary_Material.zip › supp_info_8.pdf]

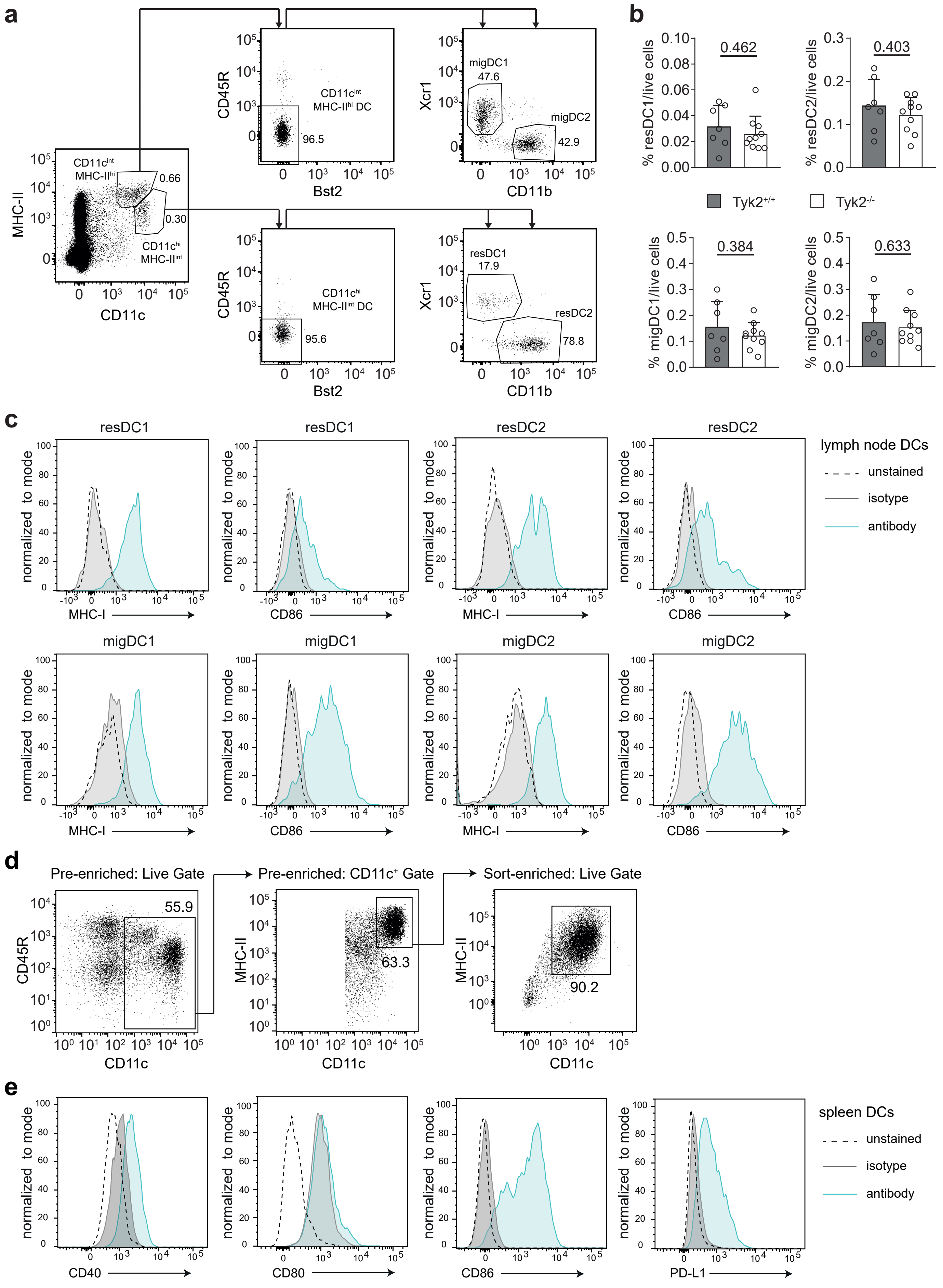
a
CD11c^int MHC-II^hi
0.66
0.30
CD11c^hi MHC-II^int
MHC-II
CD11c
CD45R
Bst2
CD11c^int MHC-II^hi DC
96.5
CD11c^hi MHC-II^int DC
95.6
Xcr1
CD11b
migDC1
47.6
migDC2
42.9
resDC1
17.9
resDC2
78.8
b
% resDC1/live cells
0.462
% resDC2/live cells
0.403
Tyk2+/+
Tyk2-/-
% migDC1/live cells
0.384
% migDC2/live cells
0.633
c
resDC1
resDC2
migDC1
migDC2
normalized to mode
MHC-I
CD86
lymph node DCs
unstained
isotype
antibody
d
Pre-enriched: Live Gate
Pre-enriched: CD11c+ Gate
Sort-enriched: Live Gate
55.9
63.3
90.2
CD45R
MHC-II
CD11c
e
normalized to mode
CD40
CD80
CD86
PD-L1
spleen DCs
unstained
isotype
antibody

Supplementary Figure 8: The number of resident and migratory cDCs is unchanged in the tumor-draining lymph nodes of metastases-bearing $TYK2^{-/-}$ host mice. (**a**) Flow cytometry gating strategy for resident and migratory cDC1 and cDC2 immune populations in tumor-draining lymph nodes of mice with liver metastases. The few pDCs were excluded by expression of CD45R which is low in cDCs and high in pDCs. (**b**) Percentage of cDCs among live (7-AAD-negative) cells in tumor-draining lymph nodes of $TYK2^{+/+}$ and $TYK2^{-/-}$ host mice. (**c**) Isotype controls for MHC-I and CD86 antibodies used to quantify the mean fluorescence intensity on cDC populations, as shown in Figure 6c, d (data were obtained by a separate experiment). (**d**) Gating strategy for enrichment and FACS sorting of splenic DCs, used for ex vivo stimulation. (**e**) Isotype controls for CD40, CD80, CD86 and PD-L1 antibodies used to quantify the mean fluorescence intensity on ex vivo stimulated DCs, as shown in Figure 6e-h. res: resident, mig: migratory. Bar diagrams represent mean values +/ - SEM with each data point representing a mouse. Flowjo software was used to analyze flow cytometry data. Statistical analysis was performed using unpaired Student's t-test. p values are indicated.
