# Supplementary material for: TYK2 Promotes Immunosurveillance of Colorectal Cancer Liver Metastasis
Source: Cancer Res. Author manuscript; Available in PMC 2025 Oct 22. (PMC7618269; doi:10.1158/0008-5472.CAN-24-4224)
Supplement: Supplementary Material [file EMS209323-supplement-Supplementary_Material.zip › supp_info_7.pdf]

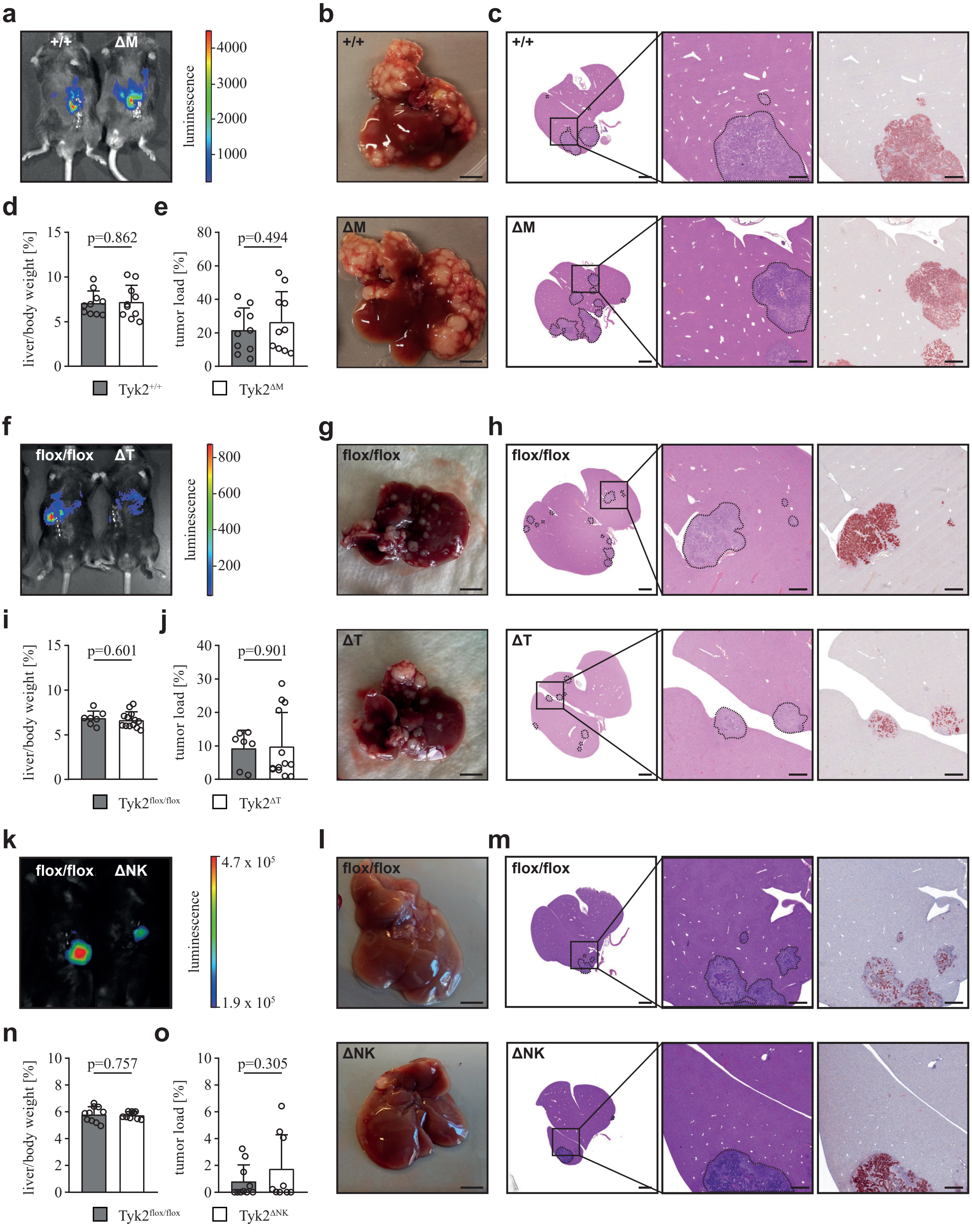
a
+/+
ΔM
luminescence
4000
3000
2000
1000
b
+/+
ΔM
c
+/+
ΔM
d
liver/body weight [%]
p=0.862
e
tumor load [%]
p=0.494
Tyk2+/+
Tyk2ΔM
f
flox/flox
ΔT
luminescence
800
600
400
200
g
flox/flox
ΔT
h
flox/flox
ΔT
i
liver/body weight [%]
p=0.601
j
tumor load [%]
p=0.901
Tyk2flox/flox
Tyk2ΔT
k
flox/flox
ΔNK
luminescence
4.7 x 10^5
1.9 x 10^5
l
flox/flox
ΔNK
m
flox/flox
ΔNK
n
liver/body weight [%]
p=0.757
o
tumor load [%]
p=0.305
Tyk2flox/flox
Tyk2ΔNK

Supplementary Figure 7: TYK2 in macrophages, granulocytes, Kupffer cells, T cells or NK cells is not required for immunosurveillance of CRLM. (**a**) Representative IVIS images of $TYK2^{+/+}$ (+/+) and $TYK2^{\Delta M}$ (ΔM) host mice, 3 weeks after intrasplenic injection of AKP organoids. (**b**) Macroscopic images of livers of $TYK2^{+/+}$ (+/+) and $TYK2^{\Delta M}$ (ΔM) host mice, 4 weeks after intrasplenic injection of AKP organoids. Scale bar = 5 mm. (**c**) H&E and GFP staining of liver sections of $TYK2^{+/+}$ (+/+, upper images) and $TYK2^{\Delta M}$ (ΔM, bottom images) host mice, 4 weeks after intrasplenic injection of AKP organoids. The images in the center represent higher magnifications of the images on the left, with the square indicating the magnified region. Dashed lines mark metastatic lesions used to quantify the tumor load shown in (e). The images on the right show immunohistochemical GFP staining of consecutive sections. Tumor cells are red. Scale bar = 2 mm for the images on the left and 500 µm for the images in the center and on the right. (**d**) Liver-to-body weight ratio of $TYK2^{+/+}$ and $TYK2^{\Delta M}$ host mice, 4 weeks after intrasplenic injection of AKP organoids. (**e**) Histomorphometric quantification of the tumor load (% of tumor area to total tissue area) of $TYK2^{+/+}$ and $TYK2^{\Delta M}$ host mice, 4 weeks after intrasplenic injection of AKP organoids. (**f**) Representative IVIS images of $TYK2^{flox/flox}$ (flox/flox) and $TYK2^{\Delta T}$ (ΔT) host mice, 3 weeks after intrasplenic injection of AKP organoids. (**g**) Macroscopic images of livers of $TYK2^{flox/flox}$ (flox/flox) and $TYK2^{\Delta T}$ (ΔT) host mice, 4 weeks after intrasplenic injection of AKP organoids. Scale bar = 5 mm. (**h**) H&E and GFP staining of liver sections of $TYK2^{flox/flox}$ (flox/flox, upper images) and $TYK2^{\Delta T}$ (ΔT, bottom images) host mice, 4 weeks after intrasplenic injection of AKP organoids. The images in the center represent higher magnifications of the images on the left, with the square indicating the magnified region. Dashed lines mark metastatic lesions used to quantify the tumor load shown in (j). The images on the right show immunohistochemical GFP staining of consecutive sections. Tumor cells are red. Scale bar = 2 mm for the images on the left and 500 µm for the images in the center and on the right. (**i**) Liver-to-body weight ratio of $TYK2^{flox/flox}$ and $TYK2^{\Delta T}$ host mice, 4 weeks after intrasplenic injection of AKP organoids. (**j**) Histomorphometric quantification of the tumor load (% of tumor area to total tissue area) of $TYK2^{flox/flox}$ and $TYK2^{\Delta T}$ host mice, 4 weeks after intrasplenic injection of AKP organoids. (**k**) Representative IVIS images of $TYK2^{flox/flox}$ (flox/flox) and $TYK2^{\Delta NK}$ (ΔNK) host mice, 3 weeks after intrasplenic injection of AKP organoids. (**l**) Macroscopic images of livers of $TYK2^{flox/flox}$ (flox/flox) and $TYK2^{\Delta NK}$ (ΔNK) host mice, 4 weeks after intrasplenic injection of AKP organoids. Scale bar = 5 mm. (**m**) H&E and GFP staining of liver sections of $TYK2^{flox/flox}$ (flox/flox, upper images) and $TYK2^{\Delta NK}$ (ΔNK, bottom images) host mice, 4 weeks after intrasplenic injection of AKP organoids. The images in the center represent higher magnifications of the images on the left, with the square indicating the magnified region. Dashed lines mark metastatic lesions used to quantify the tumor load shown in (o). The images on the right show immunohistochemical GFP staining of consecutive sections. Tumor cells are red. Scale bar = 2 mm for the images on the left and 500 µm for the images in the center and on the right. (**n**) Liver-to-body weight ratio of $TYK2^{flox/flox}$ and $TYK2^{\Delta NK}$ host mice, 4 weeks after intrasplenic injection of AKP organoids. (**o**) Histomorphometric quantification of the tumor load (% of tumor area to total tissue area) of $TYK2^{flox/flox}$ and $TYK2^{\Delta NK}$ host mice, 4 weeks after intrasplenic injection of AKP organoids. Bar diagrams represent mean values +/ - SEM with each data point representing a mouse. Case Viewer, QuPath and Halo software were used for histomorphometry. Statistical analysis was performed using unpaired Student's t-test. p values are indicated.
