# Supplementary material for: TYK2 Promotes Immunosurveillance of Colorectal Cancer Liver Metastasis
Source: Cancer Res. Author manuscript; Available in PMC 2025 Oct 22. (PMC7618269; doi:10.1158/0008-5472.CAN-24-4224)
Supplement: Supplementary Material [file EMS209323-supplement-Supplementary_Material.zip › supp_info_5.pdf]

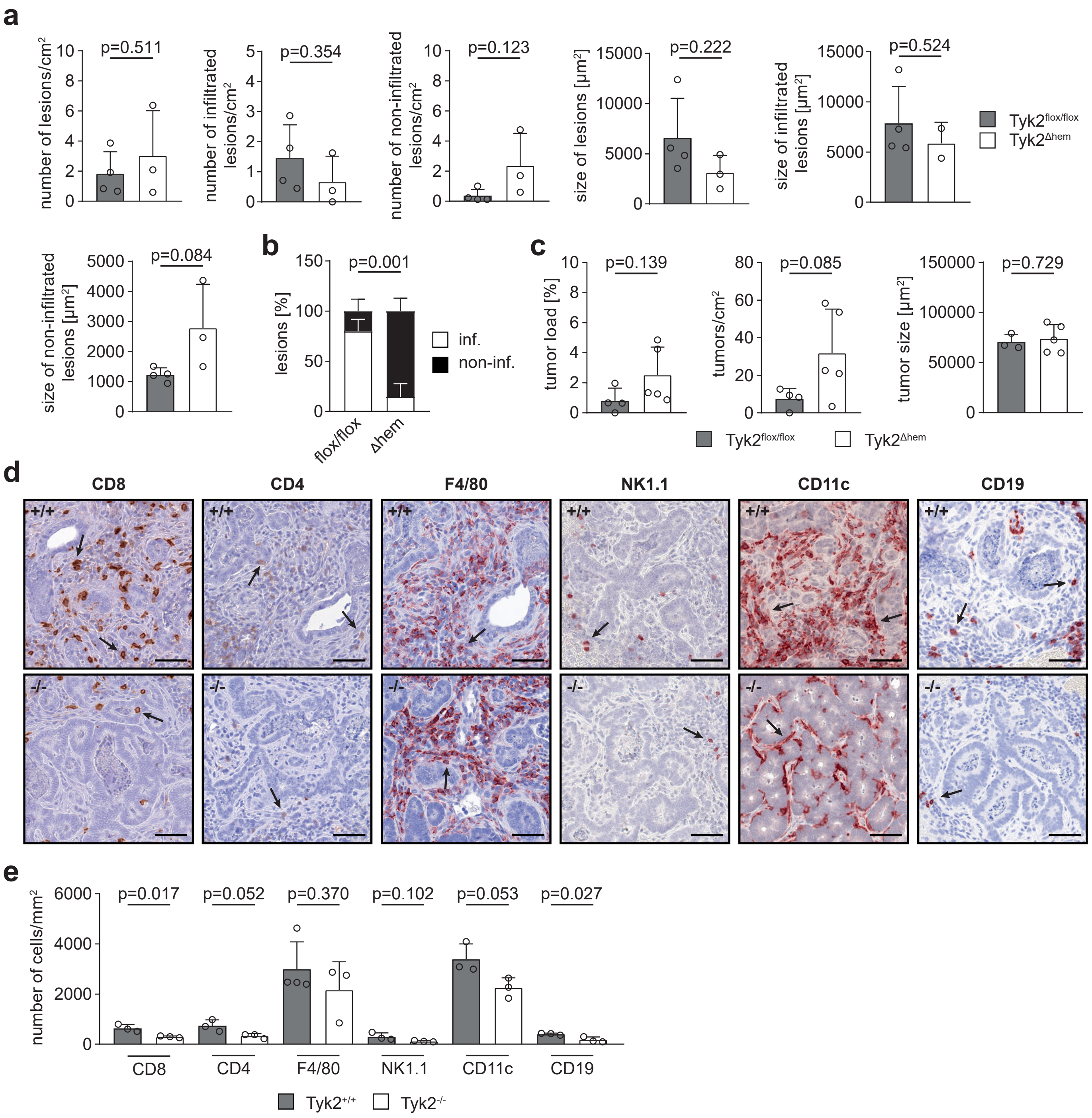

Supplementary Figure 5: Altered immunophenotype of micrometastatic lesions in TYK2$^{\Delta hem}$ host mice. (**a**) Number and size of total metastatic lesions, infiltrated metastatic lesions and non-infiltrated metastatic lesions in TYK2$^{flox/flox}$ and TYK2$^{\Delta hem}$ host mice, 7 days after intrasplenic injection of AKP organoids. (**b**) Percentage of immune-infiltrated (inf.) versus non-immune-infiltrated (non-inf.) lesions in TYK2$^{flox/flox}$ (flox/flox) and TYK2$^{\Delta hem}$ (Δhem) host mice, 7 days after intrasplenic injection of AKP organoids. (**c**) Tumor load (% of tumor area to total tissue area), tumor number and tumor size of metastasis in TYK2$^{flox/flox}$ and TYK2$^{\Delta hem}$ host mice, 14 days after intrasplenic injection of AKP organoids. (**d**) Immunohistochemical staining of CD8, CD4, F4/80, NK1.1, CD11c and CD19 in metastatic lesions of TYK2$^{+/+}$ (+/+) and TYK2$^{-/-}$ (-/-) host mice, 14 days after intrasplenic injection of AKP organoids. Positive immune cells are indicated by arrows. Scale bar = 50 µm. (**e**) Histomorphometric quantification of CD8^{+}, CD4^{+}, F4/80^{+}, NK1.1^{+}, CD11c^{+} and CD19^{+} immune cells in metastases of TYK2$^{+/+}$ and TYK2$^{-/-}$ host mice, 14 days after intrasplenic injection of AKP organoids. Bar diagrams represent mean values +/- SEM with each data point representing a mouse. Case Viewer, QuPath and Halo software were used for histomorphometry. Statistical analysis was performed using unpaired Student's t-test. p values are indicated.
