# Supplementary material for: TYK2 Promotes Immunosurveillance of Colorectal Cancer Liver Metastasis
Source: Cancer Res. Author manuscript; Available in PMC 2025 Oct 22. (PMC7618269; doi:10.1158/0008-5472.CAN-24-4224)
Supplement: Supplementary Material [file EMS209323-supplement-Supplementary_Material.zip › supp_info_4.pdf]

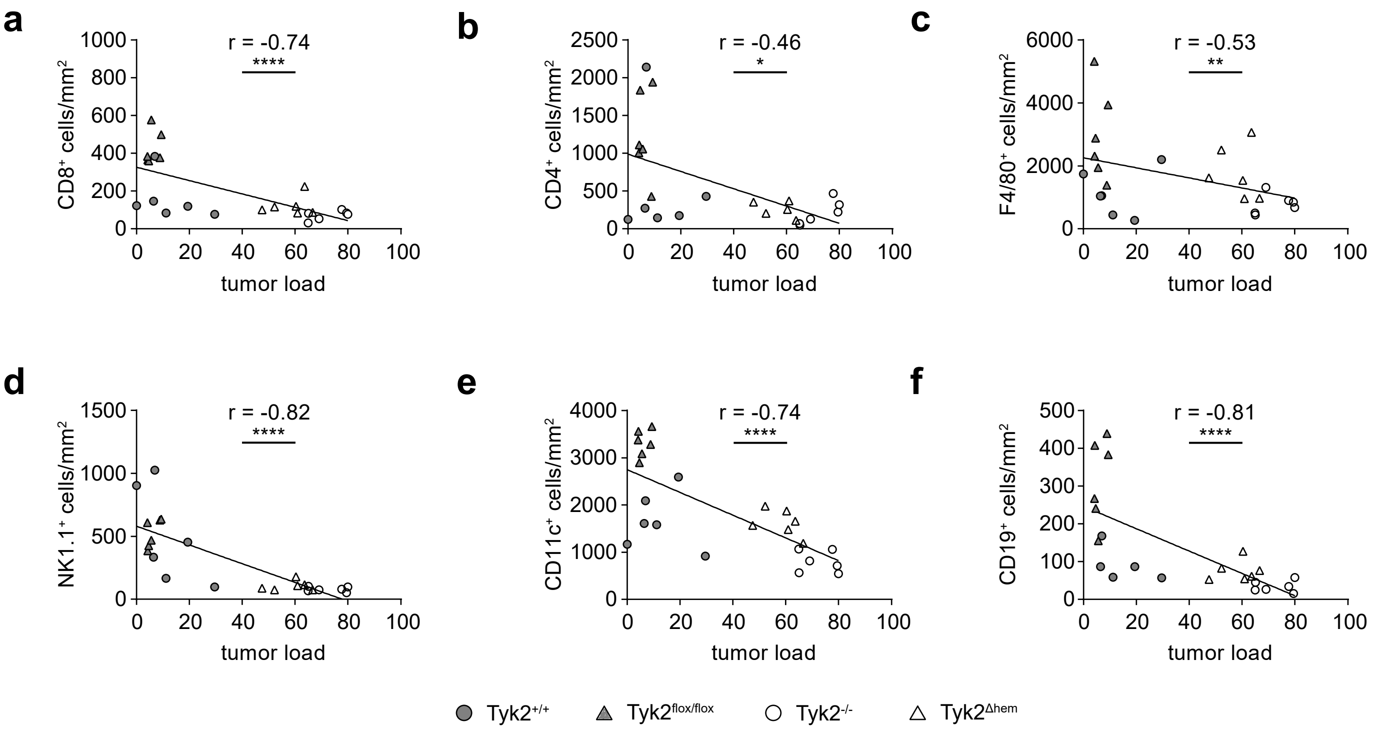

Supplementary Figure 4: Inverse correlation between metastatic load and immune infiltration. (**a-f**) Correlation analysis between the metastatic load and infiltration of specific immune cell types into metastases of TYK2-proficient and TYK2-deficient host mice, 4 weeks after intrasplenic injection of AKP organoids. (**a**) Correlation analysis for CD8^{+} cells. (**b**) Correlation analysis for CD4^{+} cells. (**c**) Correlation analysis for F4/80^{+} cells. (**d**) Correlation analysis for NK1.1^{+} cells. (**e**) Correlation analysis for CD11c^{+} cells. (**f**) Correlation analysis for CD19^{+} cells. Each data point represents a mouse. The immune infiltration data sets showed a non-normal distribution and the Spearman correlation coefficient is indicated. p values are indicated.
