# Supplementary material for: TYK2 Promotes Immunosurveillance of Colorectal Cancer Liver Metastasis
Source: Cancer Res. Author manuscript; Available in PMC 2025 Oct 22. (PMC7618269; doi:10.1158/0008-5472.CAN-24-4224)
Supplement: Supplementary Material [file EMS209323-supplement-Supplementary_Material.zip › supp_info_3.pdf]

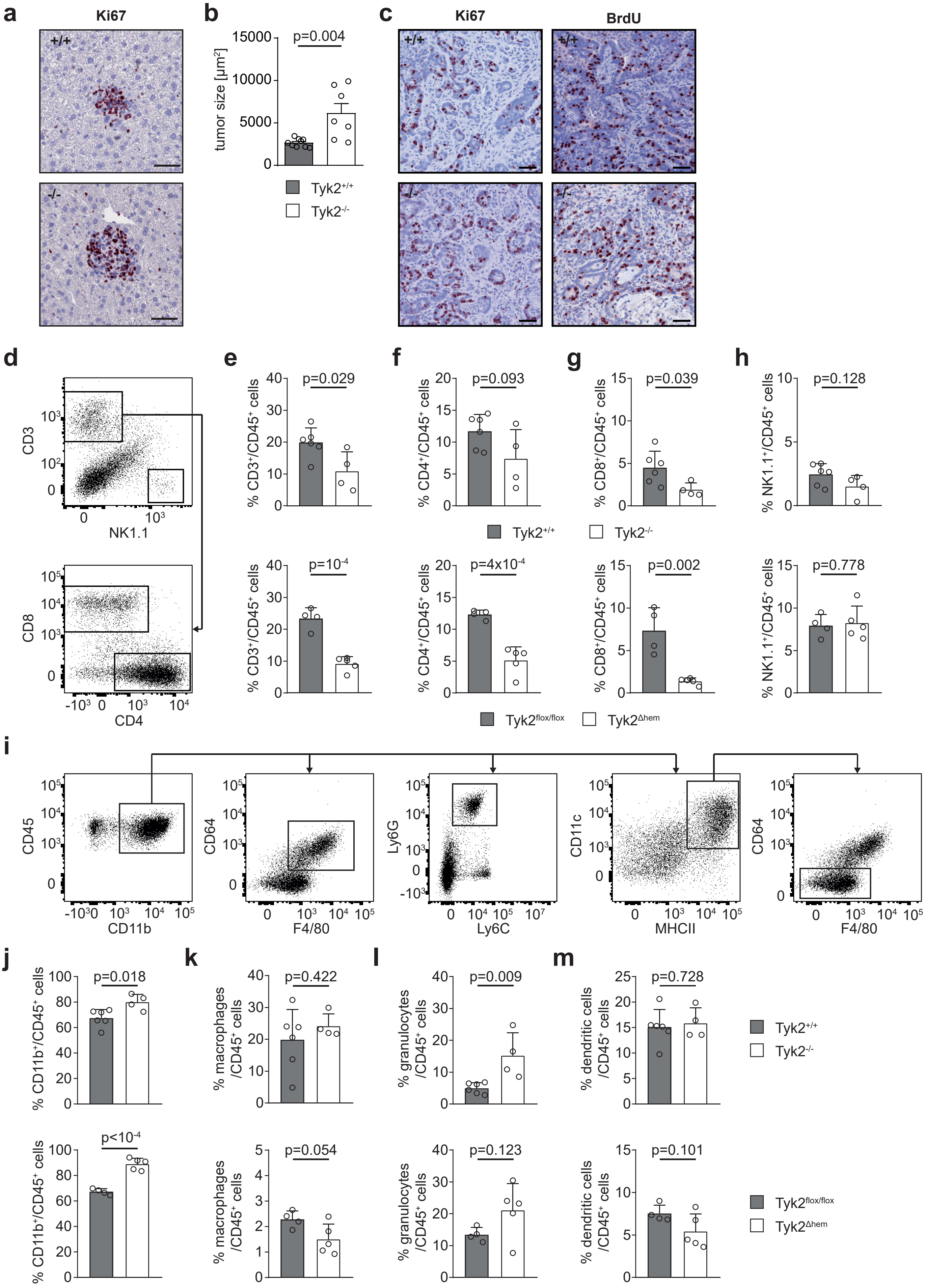

a
Ki67
+/+
-/-
b
p=0.004
tumor size [µm²]
15000
10000
5000
0
Tyk2+/+
Tyk2-/-
c
Ki67
BrdU
+/+
-/-
d
CD3
NK1.1
CD8
CD4
e
p=0.029
p=10-4
% CD3+/CD45+ cells
f
p=0.093
p=4x10-4
% CD4+/CD45+ cells
g
p=0.039
p=0.002
% CD8+/CD45+ cells
h
p=0.128
p=0.778
% NK1.1+/CD45+ cells
Tyk2+/+
Tyk2-/-
Tyk2flox/flox
Tyk2Δhem
i
CD45
CD11b
CD64
F4/80
Ly6G
Ly6C
CD11c
MHCII
CD64
F4/80
j
p=0.018
p<10-4
% CD11b+/CD45+ cells
k
p=0.422
p=0.054
% macrophages /CD45+ cells
l
p=0.009
p=0.123
% granulocytes /CD45+ cells
m
p=0.728
p=0.101
% dendritic cells /CD45+ cells
Tyk2+/+
Tyk2-/-
Tyk2flox/flox
Tyk2Δhem

Supplementary Figure 3: Immunophenotyping of established metastases by flow cytometry in TYK2$^{-/-}$ and TYK2$^{\Delta hem}$ host mice. (**a**) Representative micrometastatic lesions in TYK2$^{+/+}$ (+/+) and TYK2$^{-/-}$ (-/-) host mice, 4 weeks after intrasplenic injection of GFP-negative AKP organoids. Immunohistochemistry for the proliferation marker Ki67 was used to identify micrometastases. Scale bar = 50 µm. (**b**) Size of metastatic lesions in TYK2$^{+/+}$ (+/+) and TYK2$^{-/-}$ (-/-) host mice, 4 weeks after intrasplenic injection of GFP-negative AKP organoids (n = 6 mice per genotype, bar diagrams represent mean values +/ - SEM with each data point representing a lesion). (**c**) Immunohistochemistry for proliferation markers Ki67 and BrdU incorporation in TYK2$^{+/+}$ (+/+) and TYK2$^{-/-}$ (-/-) host mice, 4 weeks after intrasplenic injection of AKP organoids. Positive nuclei are red. Scale bar = 50 µm. (**d-m**) Flow cytometric analysis of immune infiltration in tumors of in TYK2$^{+/+}$, TYK2$^{-/-}$, TYK2$^{flox/flox}$ and TYK2$^{\Delta hem}$ host mice, 4 weeks after intrasplenic injection of AKP organoids. (**d**) Gating strategy for NK1.1^{+} and CD3^{+} immune cells. CD3^{+} NK1.1^{-} T cells were analyzed for CD4 and CD8 expression. (**e-h**) Quantification of CD3^{+}, CD4^{+}, CD8^{+} and NK1.1^{+} immune cell populations as percentage of CD45^{+} cells. Analysis of TYK2$^{+/+}$ and TYK2$^{-/-}$ metastases is shown in the upper panel, analysis of TYK2$^{flox/flox}$ and TYK2$^{\Delta hem}$ metastases is shown in the lower panel. (**i**) Gating strategy for myeloid cell populations. CD11b^{+} cells were further characterized for CD64, F4/80, Ly6C, Ly6G, CD11c and MHC-II expression. (**j-m**) Quantification of CD11b^{+} cells, macrophages (CD64^{+}, F4/80^{+}), granulocytes (Ly6G^{+}, Ly6C^{int}) and dendritic cells (CD11c^{+}, MHC-II^{+}, CD64^{-}, F4/80^{-}) as percentage of CD45^{+} cells. Analysis of TYK2$^{+/+}$ and TYK2$^{-/-}$ metastases is shown in the upper panel, analysis of TYK2$^{flox/flox}$ and TYK2$^{\Delta hem}$ metastases is shown in the lower panel. Bar diagrams represent mean values +/- SEM with each data point representing a mouse. Flowjo software was used to analyze flow cytometry data. Statistical analysis was performed using unpaired Student's t-test. p values are indicated.
