# Supplementary material for: TYK2 Promotes Immunosurveillance of Colorectal Cancer Liver Metastasis
Source: Cancer Res. Author manuscript; Available in PMC 2025 Oct 22. (PMC7618269; doi:10.1158/0008-5472.CAN-24-4224)
Supplement: Supplementary Material [file EMS209323-supplement-Supplementary_Material.zip › supp_info_2.pdf]

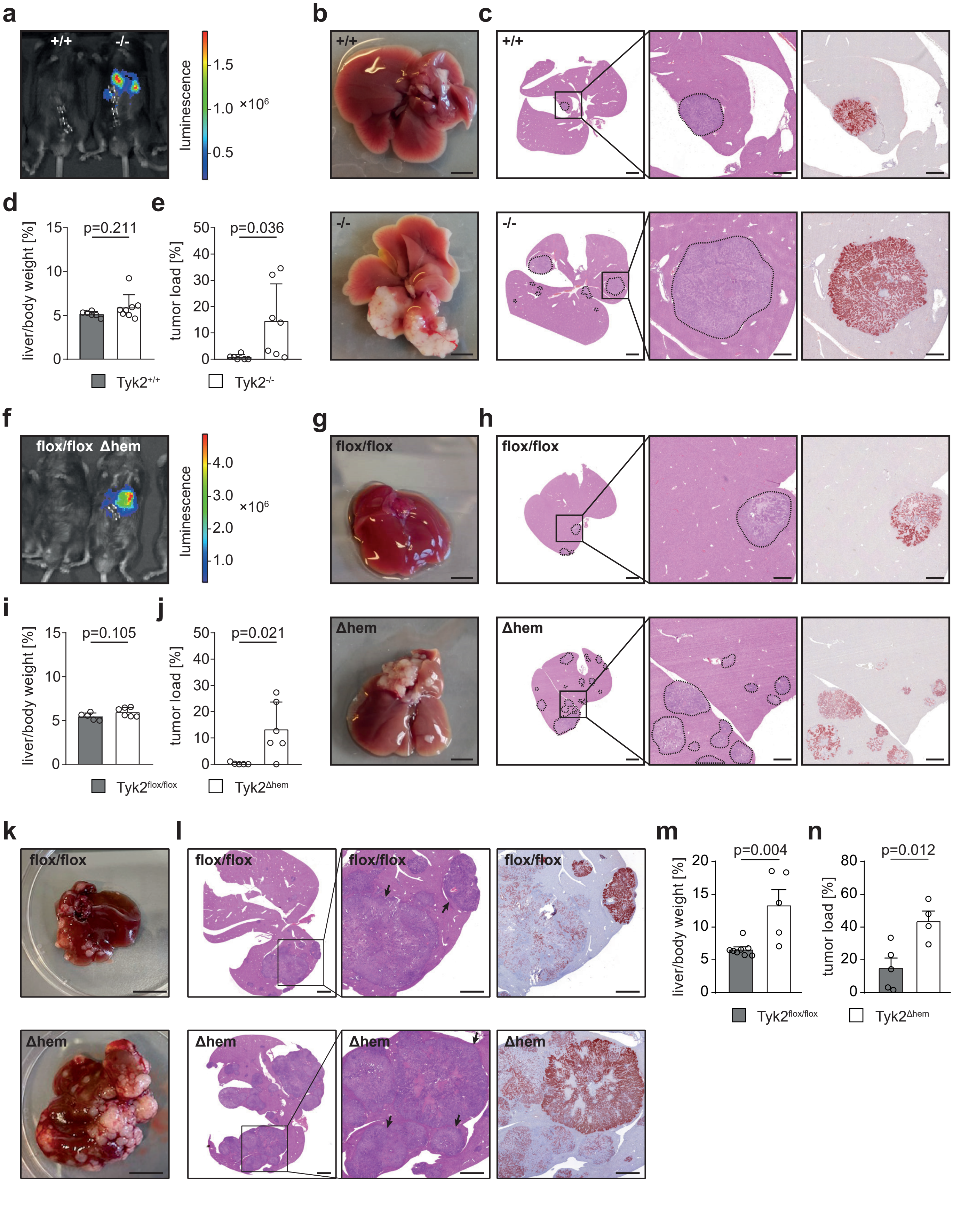

a
+/+
-/-
luminescence
1.5
1.0 ×10^6
0.5
b
c
d
liver/body weight [%]
p=0.211
e
tumor load [%]
p=0.036
Tyk2+/+
Tyk2-/-
f
flox/flox Δhem
4.0
3.0
2.0
1.0
g
flox/flox
Δhem
h
i
p=0.105
j
p=0.021
Tyk2flox/flox
Tyk2Δhem
k
l
m
p=0.004
n
p=0.012

Supplementary Figure 2: TYK2 deficiency in host mice promotes liver metastasis of AKP organoids seeded via the portal vein. (**a**) Representative IVIS images of TYK2$^{+/+}$ (+/+) and TYK2$^{-/-}$ (-/-) host mice, 3 weeks after portal vein injection of AKP organoids. (**b**) Macroscopic images of livers of TYK2$^{+/+}$ (+/+) and TYK2$^{-/-}$ (-/-) host mice, 4 weeks after portal vein injection of AKP organoids. Scale bar = 5 mm. (**c**) H&E and GFP staining of liver sections of TYK2$^{+/+}$ (+/+, upper images) and TYK2$^{-/-}$ (-/-, bottom images) host mice, 4 weeks after portal vein injection of AKP organoids. The images in the center represent higher magnifications of the images on the left, with the square indicating the magnified region. Dashed lines mark metastatic lesions used to quantify the tumor load shown in (e). The images on the right show immunohistochemical GFP staining of consecutive sections. Tumor cells are red. Scale bar = 2 mm for the images on the left and 500 µm for the images in the center and on the right. (**d**) Liver-to-body weight ratio of TYK2$^{+/+}$ and TYK2$^{-/-}$ host mice, 4 weeks after portal vein injection of AKP organoids. (**e**) Histomorphometric quantification of the tumor load (% of tumor area to total tissue area) of TYK2$^{+/+}$ and TYK2$^{-/-}$ host mice, 4 weeks after portal vein injection of AKP organoids. (**f**) Representative IVIS images of TYK2$^{flox/flox}$ (flox/flox) and TYK2$^{\Delta hem}$ (Δhem) host mice, 3 weeks after portal vein injection of AKP organoids. (**g**) Macroscopic images of livers of TYK2$^{flox/flox}$ (flox/flox) and TYK2$^{\Delta hem}$ (Δhem) host mice, 4 weeks after portal vein injection of AKP organoids. Scale bar = 5 mm. (**h**) H&E and GFP staining of liver sections of TYK2$^{flox/flox}$ (flox/flox, upper images) and TYK2$^{\Delta hem}$ (Δhem, bottom images) host mice, 4 weeks after portal vein injection of AKP organoids. The images in the center represent higher magnifications of the images on the left, with the square indicating the magnified region. Dashed lines mark metastatic lesions used to quantify the tumor load shown in (j). The images on the right show immunohistochemical GFP staining of consecutive sections. Tumor cells are red. Scale bar = 2 mm for the images on the left and 500 µm for the images in the center and on the right. (**i**) Liver-to-body weight ratio of TYK2$^{flox/flox}$ and TYK2$^{\Delta hem}$ host mice, 4 weeks after portal vein injection of AKP organoids. (**j**) Histomorphometric quantification of the tumor load (% of tumor area to total tissue area) of TYK2$^{flox/flox}$ and TYK2$^{\Delta hem}$ host mice, 4 weeks after portal vein injection of AKP organoids. (**k**) Macroscopic images of livers of TYK2$^{flox/flox}$ (flox/flox) and TYK2$^{\Delta hem}$ (Δhem) host mice, 7 weeks after intrasplenic injection of a second independent AKP organoid clone. Scale bar = 1 cm. (**l**) H&E and GFP staining of liver sections of TYK2$^{flox/flox}$ (flox/flox, upper images) and TYK2$^{\Delta hem}$ (Δhem, bottom images) host mice, 7 weeks after intrasplenic injection of a second independent AKP organoid clone. The images in the center represent higher magnifications of the images on the left, with the square indicating the magnified region. Arrows mark metastatic lesions used to quantify the tumor load shown in (n). The images on the right show immunohistochemical GFP staining of consecutive sections. Tumor cells are red. Scale bar = 2 mm for the images on the left and 1 mm for the images in the center and on the right. (**m**) Liver-to-body weight ratio of TYK2$^{flox/flox}$ and TYK2$^{\Delta hem}$ host mice, 7 weeks after intrasplenic injection of a second independent AKP organoid clone. (**n**) Histomorphometric quantification of the tumor load (% of tumor area to total tissue area) of TYK2$^{flox/flox}$ and TYK2$^{\Delta hem}$ host mice, 7 weeks after intrasplenic injection of a second independent AKP organoid clone. Bar diagrams represent mean values +/- SEM with each data point representing a mouse. Case Viewer, QuPath and Halo software were used for histomorphometry. Statistical analysis was performed using unpaired Student's t-test. p values are indicated.
