# Supplementary material for: TYK2 Promotes Immunosurveillance of Colorectal Cancer Liver Metastasis
Source: Cancer Res. Author manuscript; Available in PMC 2025 Oct 22. (PMC7618269; doi:10.1158/0008-5472.CAN-24-4224)
Supplement: Supplementary Material [file EMS209323-supplement-Supplementary_Material.zip › supp_info_1.pdf]

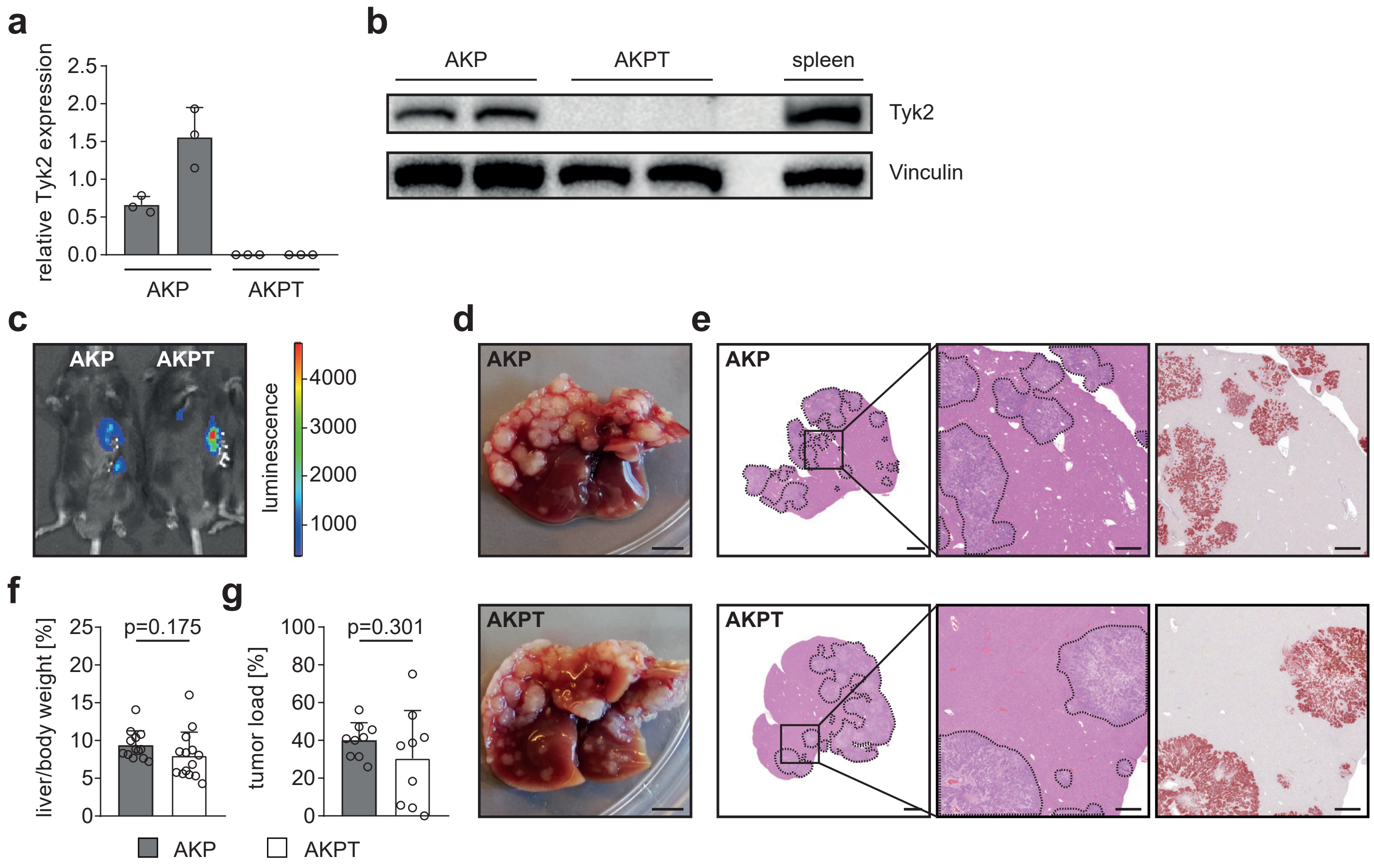

Supplementary Figure 1: *Tyk2* deletion in AKP organoids has no effect on CRLM. (**a**) Relative expression of *Tyk2* mRNA in TYK2-proficient AKP and TYK2-deficient AKPT organoids. The latter were generated by CRISPR/Cas9-mediated deletion of *Tyk2* in AKP organoids. RNA expression was analyzed by qPCR and values were normalized to expression of *Tyk2* in AKP organoids and to *Gapdh*. A bar diagram with two independent AKP and AKPT subclones is shown. (**b**) TYK2 protein expression in TYK2-proficient AKP and TYK2-deficient AKPT organoids. A Western blot with two independent AKP and AKPT subclones is shown. Protein from the spleen was included as positive control. Expression of Vinculin was used as a loading control. (**c**) Representative IVIS images of C57BL/6 host mice, 3 weeks after intrasplenic injection of AKP or AKPT organoids. (**d**) Macroscopic images of livers of C57BL/6 host mice, 4 weeks after intrasplenic injection of AKP or AKPT organoids. Scale bar = 5 mm. (**e**) H&E and GFP staining of liver sections of C57BL/6 host mice, 4 weeks after intrasplenic injection of AKP (upper images) or AKPT (bottom images) organoids. The images in the center represent higher magnifications of the images on the left, with the square indicating the magnified region. Dashed lines mark metastatic lesions used to quantify the tumor load shown in (g). The images on the right show immunohistochemical GFP staining of consecutive sections. Tumor cells are red. Scale bar = 2 mm for the images on the left and 500 µm for the images in the center and on the right. (**f**) Liver-to-body weight ratio of C57BL/6 host mice, 4 weeks after intrasplenic injection of AKP or AKPT organoids. (**g**) Histomorphometric quantification of the tumor load (% of tumor area to total tissue area) of C57BL/6 host mice, 4 weeks after intrasplenic injection of AKP or AKPT organoids. Bar diagrams represent mean values +/- SEM with each data point representing a mouse. Case Viewer, QuPath and Halo software were used for histomorphometry. Statistical analysis was performed using unpaired Student's t-test. p values are indicated.
