# Supplementary material for: TYK2 Promotes Immunosurveillance of Colorectal Cancer Liver Metastasis
Source: Cancer Res. Author manuscript; Available in PMC 2025 Oct 22. (PMC7618269; doi:10.1158/0008-5472.CAN-24-4224)
Supplement: Supplementary Material [file EMS209323-supplement-Supplementary_Material.zip › supp_info_12.pdf]

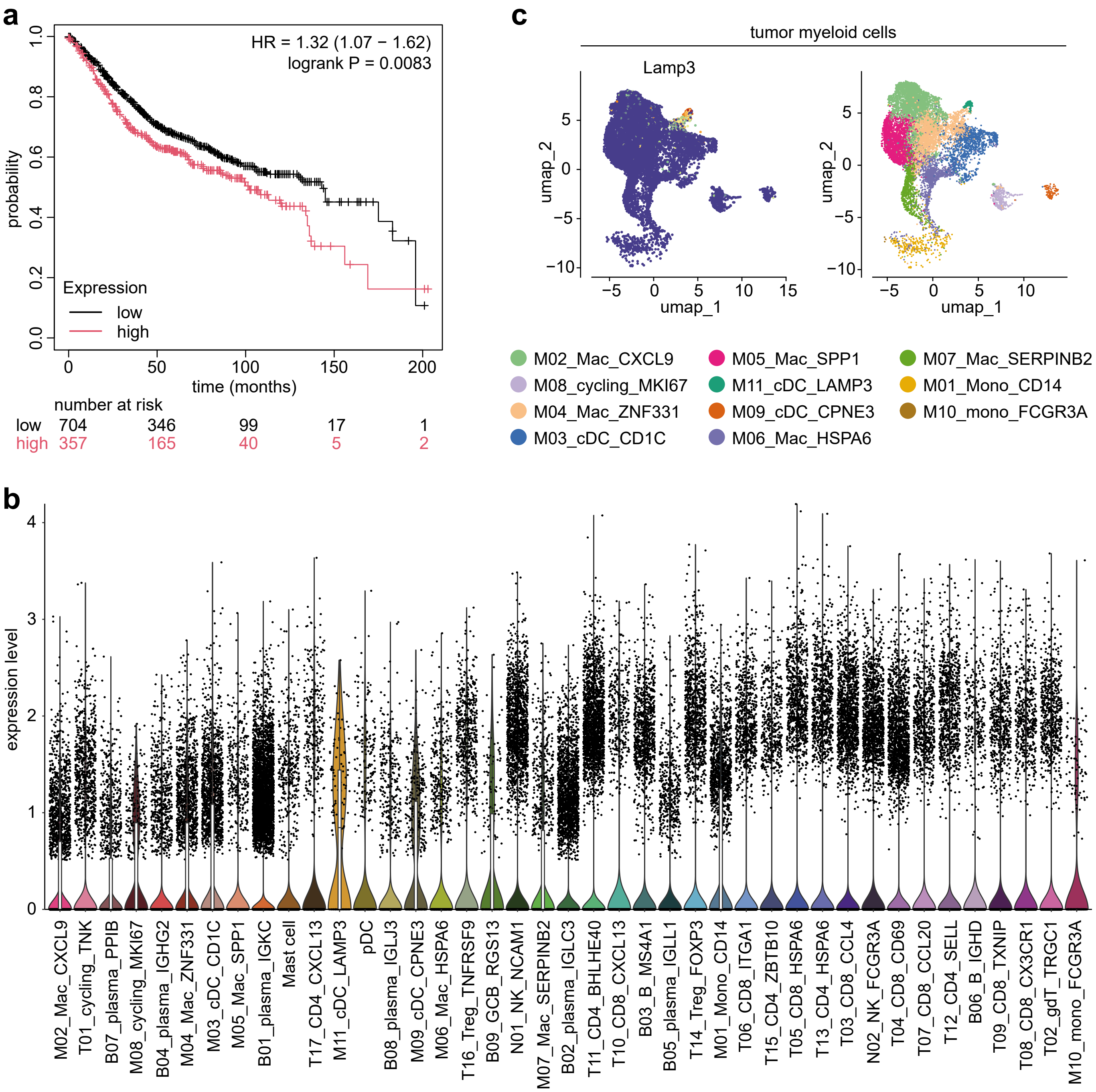

Supplementary Figure 12: Expression of TYK2 in human CRC-derived LAMP3⁺ CCR7⁺ cDCs. (**a**) Kaplan-Meier plot analysis using TCGA data from CRC patients, stratified for low and high *TYK2* mRNA expression. (**b**) Violin plots for expression of *TYK2* in immune cells from primary CRC and CRLM using a published single-cell sequencing dataset. (**c**) UMAP plots for myeloid cells in primary CRC and CRLM showing LAMP3⁺ cDCs as a distinct population.
