# Supplementary material for: TYK2 Promotes Immunosurveillance of Colorectal Cancer Liver Metastasis
Source: Cancer Res. Author manuscript; Available in PMC 2025 Oct 22. (PMC7618269; doi:10.1158/0008-5472.CAN-24-4224)
Supplement: Supplementary Material [file supp_info_10.pdf › supp_info_10.pdf]

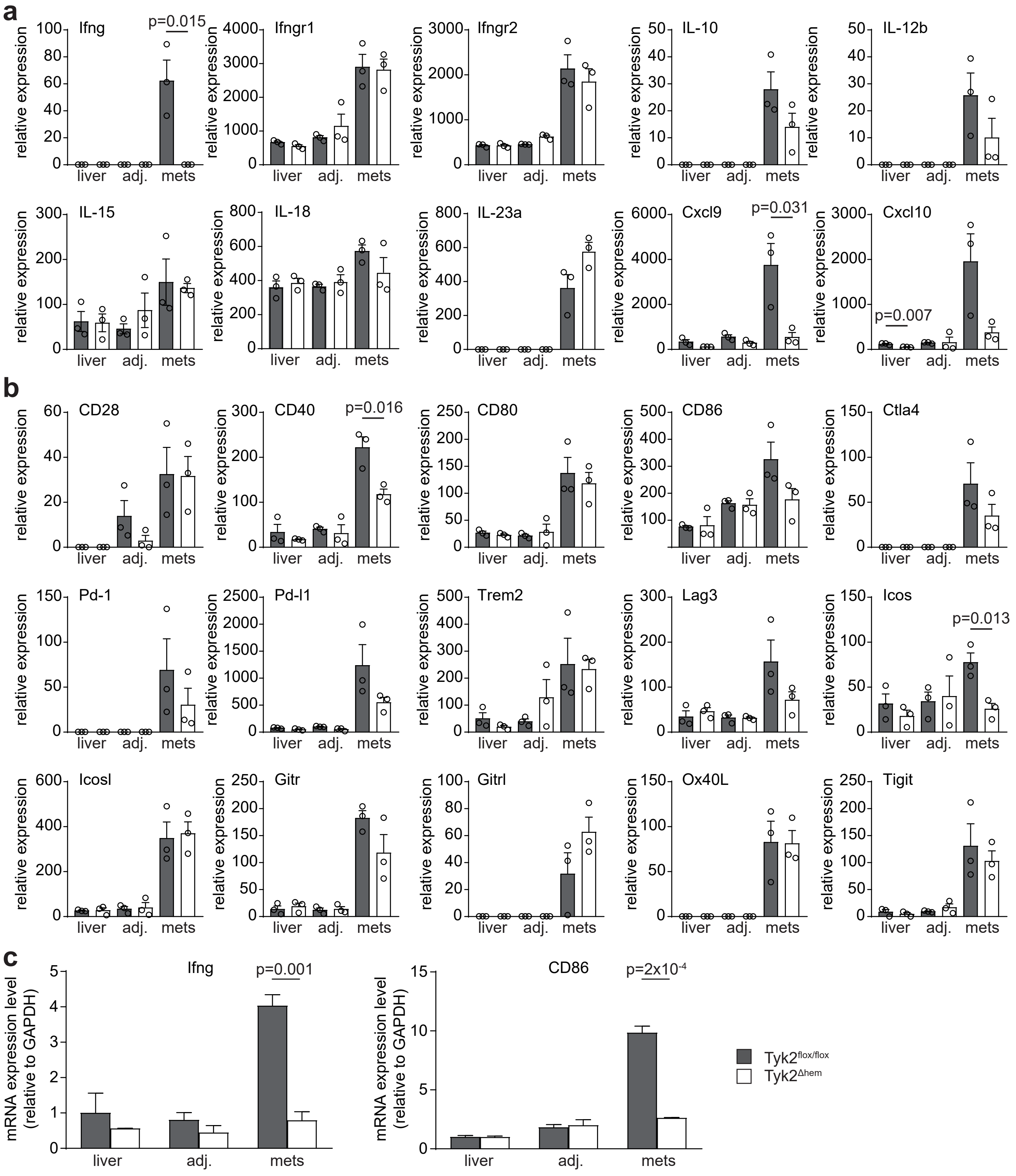

Supplementary Figure 10: mRNA expression of cytokines, chemokines, costimulatory molecules and immune checkpoints in healthy and metastases-bearing livers of $TYK2^{flox/flox}$ and $TYK2^{\Delta hem}$ host mice. (**a**) mRNA expression of indicated cytokines and chemokines as well as receptors for IFN-γ in healthy and metastases-bearing livers of $TYK2^{flox/flox}$ and $TYK2^{\Delta hem}$ host mice. (**b**) mRNA expression of indicated costimulatory molecules and immune checkpoints in healthy and metastases-bearing livers of $TYK2^{flox/flox}$ and $TYK2^{\Delta hem}$ host mice. Relative expression values in (a) and (b) were derived from bulk RNA sequencing data. (**c**) qPCR analysis for expression of *Ifn-γ* and *CD86* in healthy and metastases-bearing livers of $TYK2^{flox/flox}$ and $TYK2^{\Delta hem}$ host mice (n = 3 mice per genotype). liver: healthy liver without metastases, adj.: liver tissue adjacent to metastatic lesions, mets: tissue from isolated metastases. Bar diagrams represent mean values +/- SEM with each data point representing a mouse. Statistical analysis was performed using unpaired Student's t-test. p values are indicated.
